# Mitochondria are transported along microtubules in membrane nanotubes to rescue distressed cardiomyocytes from apoptosis

**DOI:** 10.1038/s41419-017-0145-x

**Published:** 2018-01-23

**Authors:** Jing Shen, Jiang-Hui Zhang, Han Xiao, Ji-Min Wu, Kang-Min He, Zhi-Zhen Lv, Zi-Jian Li, Ming Xu, You-Yi Zhang

**Affiliations:** 1 Department of Cardiology and Institute of Vascular Medicine, Peking University Third Hospital, Beijing, 100191 China; 2 Key Laboratory of Cardiovascular Molecular Biology and Regulatory Peptides, Ministry of Health, Beijing, 100191 China; 3 Key Laboratory of Molecular Cardiovascular Science, Ministry of Education, Beijing, 100191 China; 4 Beijing Key Laboratory of Cardiovascular Receptors Research, Beijing, 100191 China

## Abstract

Membrane nanotubes (MNTs) act as “highways” between cells to facilitate the transfer of multiple signals and play an important role in many diseases. Our previous work reported on the transfer of mitochondria via MNTs between cardiomyocytes (CMs) and cardiac myofibroblasts (MFs); however, the elucidation of the underlying mechanism and pathophysiological significance of this transfer requires additional study. In this study, we determined that the mean movement velocity of mitochondria in MNTs between CMs and MFs was approximately 17.5 ± 2.1 nm/s. Meanwhile, treatment with microtubule polymerisation inhibitors nocodazole or colcemid in cell culture decreased mitochondrial velocity, and knockdown of the microtubule motor protein kinesin family member 5B (KIF5B) led to a similar effect, indicating that mitochondrial movement was dependent on microtubules and the motor protein KIF5B. Furthermore, we showed that hypoxia/reoxygenation-induced CM apoptosis was attenuated by coculture with intact or hypoxia/reoxygenation-treated MFs, which transferred mitochondria to CMs. This rescue was prevented either by separating the cells using Transwell culture or by impairing mitochondrial transfer with nocodazole or colcemid treatment. In conclusion, as a novel means of intercellular communication, MNTs rescue distressed CMs from apoptosis by transporting mitochondria along microtubules via KIF5B.

## Introduction

The intimate communication between cardiomyocytes (CMs) and cardiac fibroblasts is very important for the maintenance of normal myocardial function, and it affects the outcomes of heart disease therapies^1,2^. Membrane nanotubes (MNTs) are newly identified long, thin, membrane-based distant connections^3^ that facilitate the exchange of diverse cargoes^4,5^. In 2010, our group first reported that MNTs were formed in rat CM-derived H9c2 cells and transferred quantum dots^6^. Furthermore, we described the formation of MNTs between CMs and myofibroblasts (MFs); these structures are involved in the intercellular transfer of Ca^2+^ and mitochondria^7^. The presence of MNTs, which weave a functional network among different types of cells, extends beyond the traditional view of intracellular communication between CMs and cardiac fibroblasts^8^. However, the mechanism and physiological relevance of mitochondrial transport in MNTs between CMs and MFs remain unknown.

Mitochondria are essential for energy production and play a key role in modulating apoptosis^9–11^. Mitochondrial dysfunction is a crucial determinant of myocyte loss during ischaemia and is an important mechanism in the pathogenesis of ischaemia/reperfusion injury and cardiomyopathy^12^. Recent studies have shown that the mitochondrial transfer between cells can rescue injured cells^13,14^. Long et al.^15^ showed that CMs are more vulnerable than cardiac fibroblasts to hypoxia exposure. Therefore, we hypothesised that mitochondria can be transferred in MNTs between distant intact MFs and distressed CMs upon hypoxia/reoxygenation injury and subsequently rescue distressed CMs from apoptosis.

In the present study, we characterised mitochondrial motion in MNTs and identified the underlying mechanism. Furthermore, we investigated apoptosis in hypoxia/reoxygenation (H/R)-treated CMs after coculture with MFs. Additionally, we identified an important role for MNT-mediated mitochondrial transfer in the protection of distressed CMs by MFs. Our study elucidated the mechanism and physiological relevance of mitochondrial transfer in MNTs between CMs and MFs.

## Results

### Mobility of mitochondria in MNTs between neonatal rat ventricular CMs and MFs

**Fig. 1:**
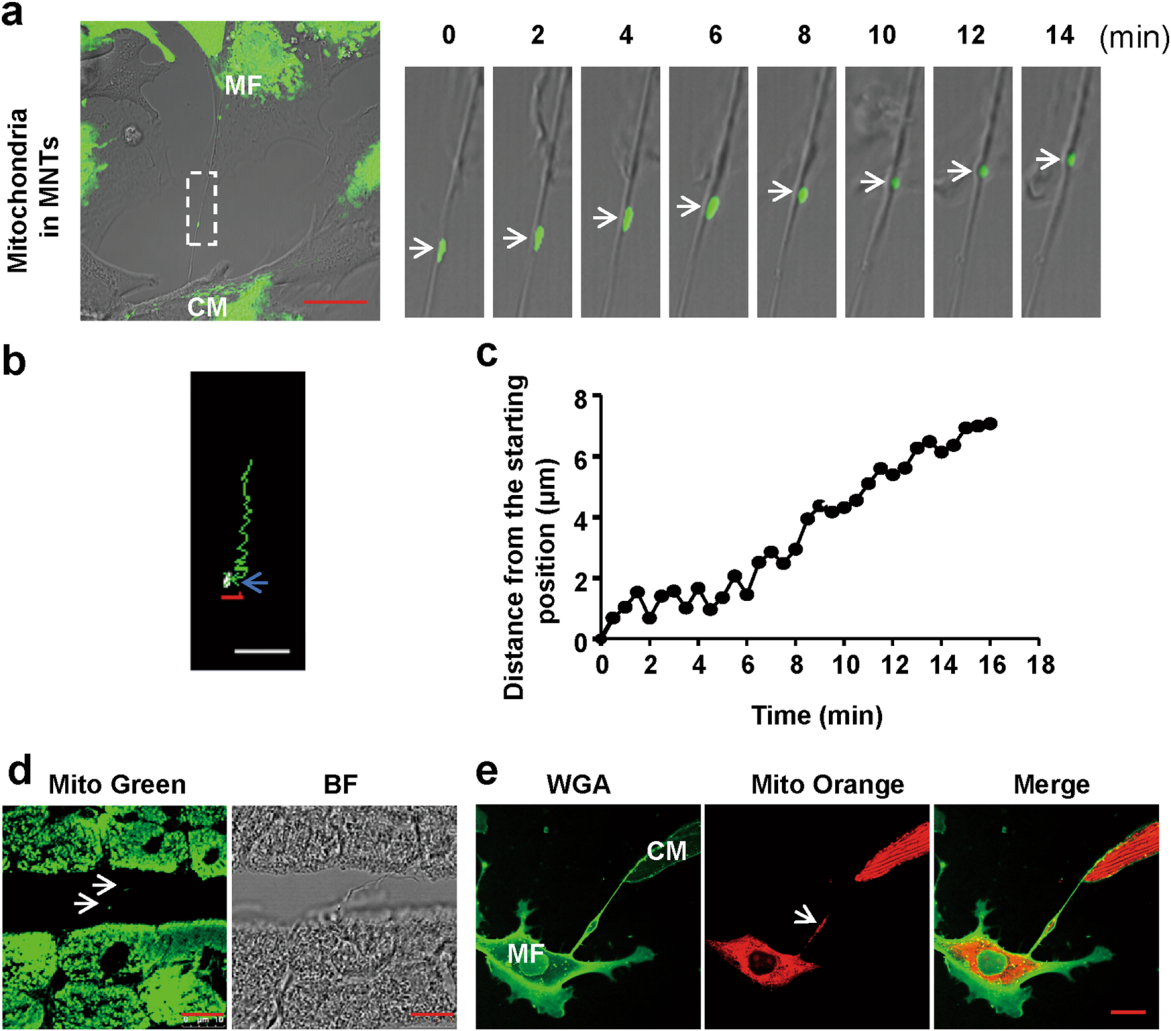
Mitochondrial movement in MNTs between neonatal rat ventricular CMs and MFs **a** A series of confocal images from a time-lapse movie (Online supplementary material, movie 1) show the movement of the EGFP-labelled mitochondrion (Ad-Mito-EGFP, arrows) between two cells via a MNT. Scale bar: 20 μm. **b** The mobile trajectory of a representative mitochondrion in a MNT in (**a**). The arrow points to the start point, and the red number “1” was automatically generated by the analysis software to specify the analysed mitochondrion. Scale bar: 5 μm. **c** Distance changes over time (relative to the starting point) of the mitochondrion in (**a**). **d** Confocal micrographs of adult rat heart frozen sections stained with MitoTracker Green (Mito Green). White arrows indicate the mitochondria within MNTs. BF, bright field. *N* = 5. Scale bar: 10 μm. **e** Cocultured adult CMs and MFs. Membrane and mitochondria were labelled by Alexa Fluor 488-conjugated WGA (WGA, green) and MitoTracker Orange (Mito Orange), respectively. White arrows indicate the mitochondria within the MNTs. Scale bar: 20 μm

Using live cell imaging, we first tracked the movement and analysed the motion characteristics of mitochondria in MNTs. Because mitochondrial fluorescent dye is easily bleached, we constructed adenoviral-mitochondria-enhanced green fluorescent protein (Ad-Mito-EGFP), which contained a mitochondrial targeting sequence (Supplementary Figure S1a). Ad-Mito-EGFP allowed us to track mitochondria over longer periods of time. To determine whether the Ad-Mito-EGFP-labelled structures were indeed mitochondria, the pattern of EGFP fluorescence was compared with that of orange-fluorescent mitochondrial dye (Mito Orange) staining. The results showed co-localisation between EGFP and the orange dye, indicating that Ad-Mito-EGFP could specifically label mitochondria in CMs and MFs (Supplementary Figure S1b). In the CM and MF coculture system, Ad-Mito-EGFP-labelled mitochondria within the MNTs between adjacent cells were tracked by time-lapse confocal microscopy. Several mitochondria showed active and saltatory movements (abrupt movements) in MNTs (Fig. 1a; Supplementary Material, Movie 1), with a mean velocity of approximately 17.5 ± 2.1 nm/s and a maximum velocity of up to 256.0 nm/s (*n* = 12 mitochondria in 6 MNTs). The mobile trajectories and distances of mitochondrial movement at different time points are shown in Fig. 1b, c. Moreover, mitochondria stained by MitoTracker were also observed in MNTs between cardiac cells in heart tissue or between adult cardiomyocytes and adult cardiac fibroblasts (Fig. 1d, e).

### Microtubules are required to transfer mitochondria in MNTs

**Fig. 2:**
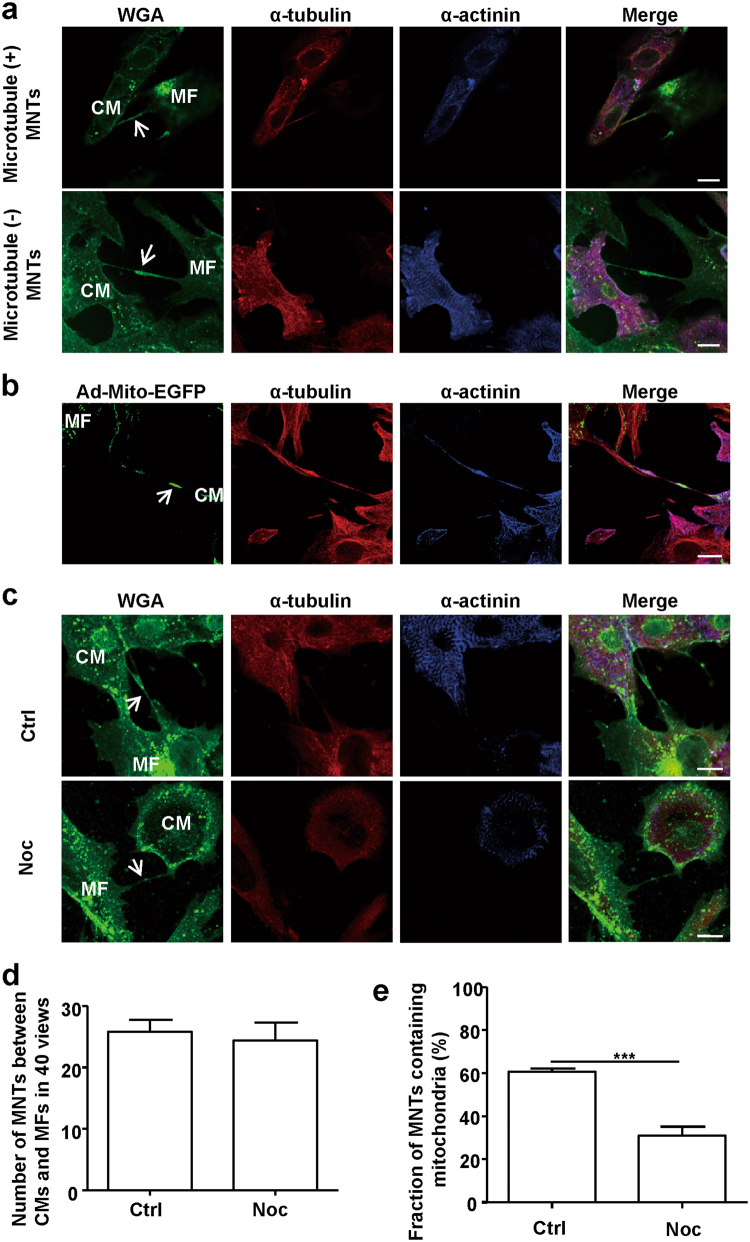
Microtubules are required for mitochondrial localisation in MNTs between CMs and MFs ** a** Cocultured CM and MF membranes were labelled by WGA (green), microtubules were labelled by α-tubulin antibodies (red) and CMs were labelled by CM-specific sarcomeric α-actinin antibodies (blue). Arrows indicate MNTs with (+) and without (−) microtubules. *N* = 5. Scale bar: 10 μm. **b** Cocultured CMs and MFs were transfected with Ad-Mito-EGFP (green) and then stained with antibodies against α-tubulin (red) and α-actinin (blue). Microtubules were observed in all mitochondria-containing MNTs (*n* = 36 from 3 independent experiments). The arrow points to a mitochondrion in the MNT. Scale bar: 20 μm. **c** Cocultured CMs and MFs were triple labelled by WGA, α-tubulin and α-actinin and then treated with or without the microtubule polymerisation inhibitor nocodazole (Noc). WGA-labelled MNTs were still detectable (arrows) in the cells treated with nocodazole. Ctrl, control. Scale bar: 10 μm. **d** Quantification of 40 randomly selected fields showing a difference in the number of MNTs between CMs and MFs in the control and nocodazole treatment groups (*N* = 5). **e** A fraction of mitochondria-containing MNTs between CMs and MFs was significantly decreased when the cells were treated with nocodazole (*N* = 5). Data are shown as the mean ± S.E.M. ***P < 0.001 using Student’s unpaired two-tailed *t*-test

We further investigated the mechanism of mitochondrial transfer in MNTs. In axons, mitochondria are transferred along microtubules and actin microfilaments^16^. Specifically, the long-range transport of mitochondria is dependent on microtubules^17^. Previously, we reported that both F-actin and microtubules existed in MNTs between CMs and MFs^7^. Therefore, we investigated whether microtubules or F-actin were required for mitochondrial movement in MNTs. First, microtubules were only observed in a fraction of MNTs between CMs and MFs (Fig. 2a). Upon examining 54 randomly selected MNTs, we found that approximately 54 ± 12% of the MNTs contained microtubules, and 60 ± 8% of the MNTs contained mitochondria. In contrast, all randomly selected mitochondria-containing MNTs contained microtubules (Fig. 2b). When microtubules were disrupted by nocodazole, an inhibitor of microtubule polymerisation, MNT formation between CMs and MFs was not affected (Fig. 2c, d). However, the ratio of mitochondria-containing MNTs decreased significantly (Fig. 2e). These results indicate that microtubules are required for mitochondria localisation in MNTs. Unlike microtubules, F-actin was detected in all randomly selected MNTs (*n* = 44). In addition, MNTs were disrupted when F-actin was destroyed by cytochalasin D, an inhibitor of actin polymerisation (Supplementary Figure S2a). Thus, F-actin may be an integral structural component of MNTs. The specific effects of cytochalasin D and nocodazole were confirmed by simultaneously staining microtubules and F-actin (Supplementary Figure S2b-c).

**Fig. 3:**
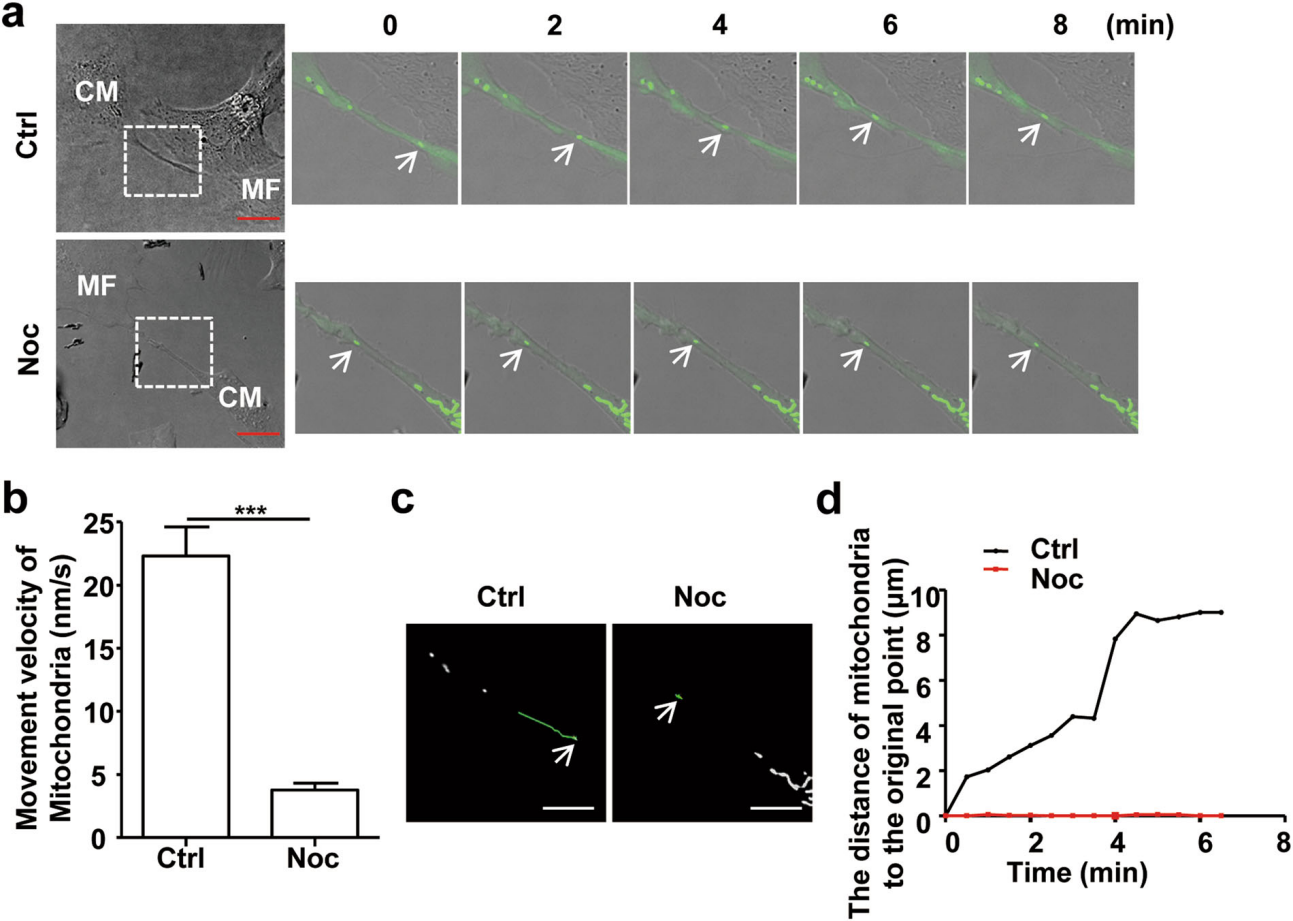
Microtubules are essential for mitochondrial movement in MNTs between CMs and MFs **a** A series of confocal images from time-lapse movies (Online supplementary material, movies 2 and 3) show the movement of Ad-Mito-EGFP-labelled mitochondria (arrows) in MNTs between CMs and MFs, with or without nocodazole treatment. Scale bar: 20 μm. **b** Mean movement velocities of mobile mitochondria in MNTs, with or without nocodazole treatment (*n* = 13 mitochondria from 3 independent experiments). **c** The mobile trajectories of mitochondria in MNTs (in (**a**)) were shorter after nocodazole treatment. The trajectories are represented by green lines, and the arrows point to the starting points. Scale bar: 7.5 μm. **d** Distance changes over time (relative to the starting point) of the mitochondria are shown in (**a**). Data are shown as the mean ± S.E.M. ***P < 0.001 using Welch’s *t*-test

To further confirm the role of microtubules in mitochondrial transport in MNTs, we examined the effects of nocodazole on mitochondrial movement in MNTs. We found that mitochondrial mobility was dramatically suppressed and that the velocity decreased from 22.3 ± 2.4 nm/s to 3.8 ± 0.5 nm/s when the microtubule structures were disrupted by nocodazole (Fig. 3a, b; Supplementary Movies 2-3). The time-dependent changes in mobile trajectory and distance were also decreased (Fig. 3c, d). Another microtubule depolymerisation reagent, colcemid, played a role similar to that of nocodazole. Colcemid significantly decreased mitochondrial movement velocity but did not affect the formation of MNTs (Supplementary Figure S3). Together, these results suggest that microtubules are required for mitochondrial movement in MNTs between CMs and MFs.

### Kinesin family member 5B (KIF5B) is required for mitochondrial transfer in MNTs

**Fig. 4:**
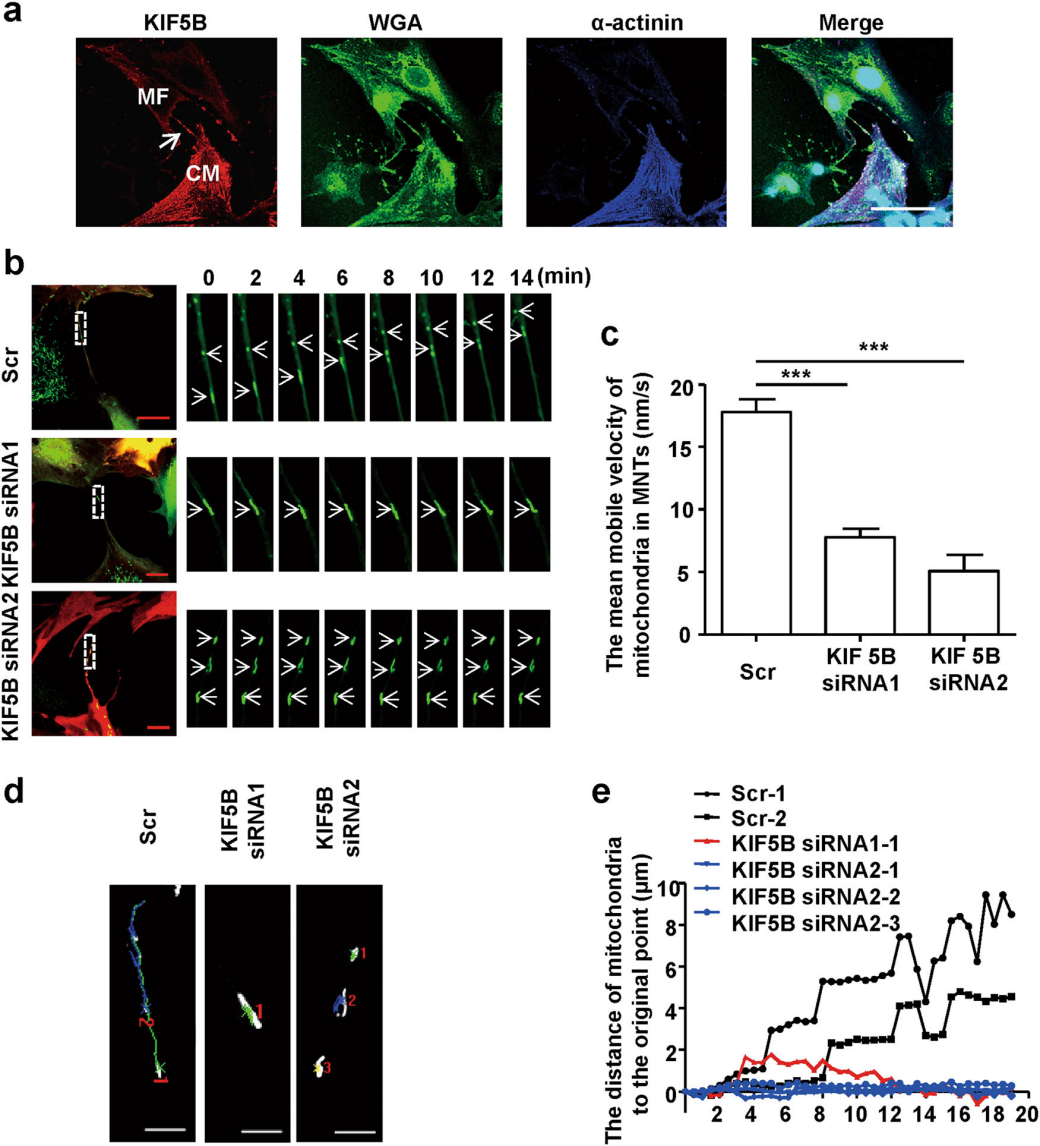
KIF5B is the motor protein involved in the transfer of mitochondria in MNTs **a** Cocultured CMs and MFs were triple labelled for KIF5B (red), WGA (membrane marker, green) and α-actinin (CM marker, blue). KIF5B was observed in CMs and MFs, as well as in MNTs between the two cell types (arrow). Scale bar: 50 μm. **b** A series of confocal images from the time-lapse movies (Online supplementary material, movies 4-6) show the movement of Ad-Mito-EGFP-labelled mitochondria in MNTs between cells treated with scramble siRNA (Scr) or KIF5B siRNA1/KIF5B siRNA2. RFP-positive cells (red) indicate the successful transfection of lentivirus carrying siRNA, and EGFP-positive bodies (green) are mitochondria. Scale bar: 20 μm. **c** Quantification of the mean movement velocities of Ad-Mito-EGFP-labelled mitochondria in MNTs in cells, with or without KIF5B depletion (Scr: *n* = 7 mitochondria; siRNA1: *n* = 5 mitochondria; and siRNA2: *n* = 13 mitochondria, from 3 independent experiments). **d** The mobile trajectories of mitochondria in MNTs (in (**b**)) were shorter after KIF5B depletion. The trajectories from different mitochondria are represented as different coloured lines, and the red number was automatically generated by the analysis software to specify the mitochondrion analysed. Scale bar: 5 μm. **e** Distance changes over time (relative to the original point) of mitochondria are shown in (**b**). Data are shown as the mean ± S.E.M. *** *P* < 0. 001 using one-way ANOVA with Tukey’s post hoc test

Kinesins constitute a large superfamily of motor proteins and are believed to be the major proteins involved in cell periphery-directed transport processes along microtubules^18^. Several reports have associated KIF3B, KIF5A, KIF5B and KIF5C, all of which belong to the kinesin superfamily, with the transfer of mitochondria^19^. Therefore, we examined the expression levels of these motor proteins in CMs and MFs by real-time PCR (brain tissue was used as a positive control). We found that of the examined kinesins, only KIF5B was expressed at a high level (Supplementary Figure S4a). We also confirmed the expression of KIF5B in CMs and MFs and the existence of KIF5B in MNTs by immunostaining (Fig. 4a). These findings suggest that KIF5B is likely to be the motor protein involved in mitochondrial transfer within MNTs.

To confirm that KIF5B is involved in the transfer of mitochondria in MNTs, lentiviral vectors containing KIF5B small interfering RNA (siRNA; labelled by red fluorescent protein (RFP)) were used to deplete KIF5B expression (Supplementary Figure S4b). RFP fluorescence was observed in most CMs and MFs 5 days after lentiviral transduction, indicating successful infection (Supplementary Figure S4c). Effective depletion of KIF5B expression was further confirmed by western blot analysis (Supplementary Figure S5). We have determined that microtubules are required for mitochondrial movement in MNTs (Figs. 2 and 3); therefore, we visualised microtubular structures in the KIF5B-depleted cells. As shown in Supplementary Figure S6, microtubules were not affected by KIF5B depletion. However, the mobility of mitochondria in MNTs was significantly inhibited when KIF5B was depleted (V_NC = _17.8 ± 1.0 nm/s; V_KIF5B siRNA1_ = 7.8 ± 0.7 nm/s and V_KIF5B siRNA2_ = 5.1 ± 1.3 nm/s) (Fig. 4b and c; Supplementary Movies 4-6). The changes in the mobile trajectory and distances over time were also decreased (Fig. 4d and e). These data indicate that the motor protein KIF5B is involved in mitochondrial movement in MNTs.

### MNT-mediated transfer of mitochondria from MFs rescues distressed CMs from apoptosis

**Fig. 5:**
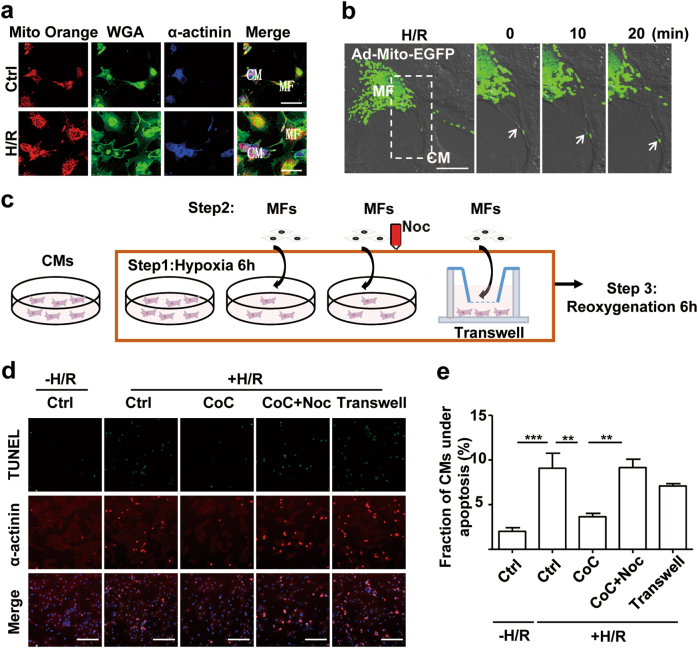
H/R-induced CM apoptosis can be rescued by the MNT-mediated transfer of mitochondria from intact MFs **a** With or without prior hypoxia treatment for 6 h, CMs were cocultured with Mito Orange (red)-labelled MFs for 6 h under normoxic conditions. Cocultured CMs and MFs were stained with WGA (membrane marker, green) and α-actinin (CM marker, blue). Mito Orange staining was observed in CMs, suggesting mitochondrial transfer from MFs to CMs. Scale bar: 50 μm. **b** CMs treated with hypoxia for 6 h were then cocultured with Ad-Mito-EGFP-transfected MFs. A series of confocal images show the transfer of mitochondria (arrows) from MFs to CMs within MNTs. Scale bar: 10 μm. **c** Schematic representation of the cell treatments. **d** H/R-induced CM apoptosis was identified by TUNEL staining (green) and high-content screening imaging. CMs were differentiated by α-actinin staining (red). CoC, coculture. Scale bar: 100 μm. **e** Quantification of the fraction of apoptotic CMs by high-content screening (*N* = 5). Data are shown as the mean ± S.E.M. **P < 0. 01 and ***P < 0. 001 using one-way ANOVA with Tukey’s post hoc test

To identify the physiological relevance of mitochondrial transfer within MNTs, we studied the effect of MNTs on CM apoptosis after H/R exposure. As shown in Figure S7, MNTs started to form between CMs and MFs after 4 h of coculture, with or without hypoxia exposure (Supplementary Figure S7). The number of MNTs between CMs and MFs was nearly identical between the normoxia and hypoxia treatment groups (Supplementary Figure S7). Next, we investigated whether the mitochondria in MFs could be transported into CMs following H/R treatment. When Mito Orange-labelled MFs were cocultured with CMs for 6 h, Mito Orange dye could be observed in several CMs by confocal microscopy (Fig. 5a). Using live cell imaging, Ad-Mito-EGFP-labelled mitochondria were observed to move from MFs to CMs within MNTs (Fig. 5b; Supplementary Movie 7). Thus, mitochondria from MFs could be transported into CMs. Using transferase dUTP nick-end labelling (TUNEL) staining, we found that the occurrence of apoptosis in distressed CMs decreased when cocultured with intact MFs. To determine the role of mitochondrial transfer within MNTs, CMs and MFs were treated as shown in Fig. 5c. The rescue effect was not observed under Transwell culture conditions or when the microtubules were disrupted by nocodazole or colcemid (Fig. 5d and e; Supplementary Figure S8 a-c). Transwell culture conditions blocked the formation of MNTs between CMs and MFs, and the destruction of the microtubules inhibited mitochondrial movement in MNTs (Figs. 2 and 3). These results suggest that apoptosis induced by H/R in CMs can be attenuated by intact MFs via the transport of mitochondria through MNTs.

**Fig. 6:**
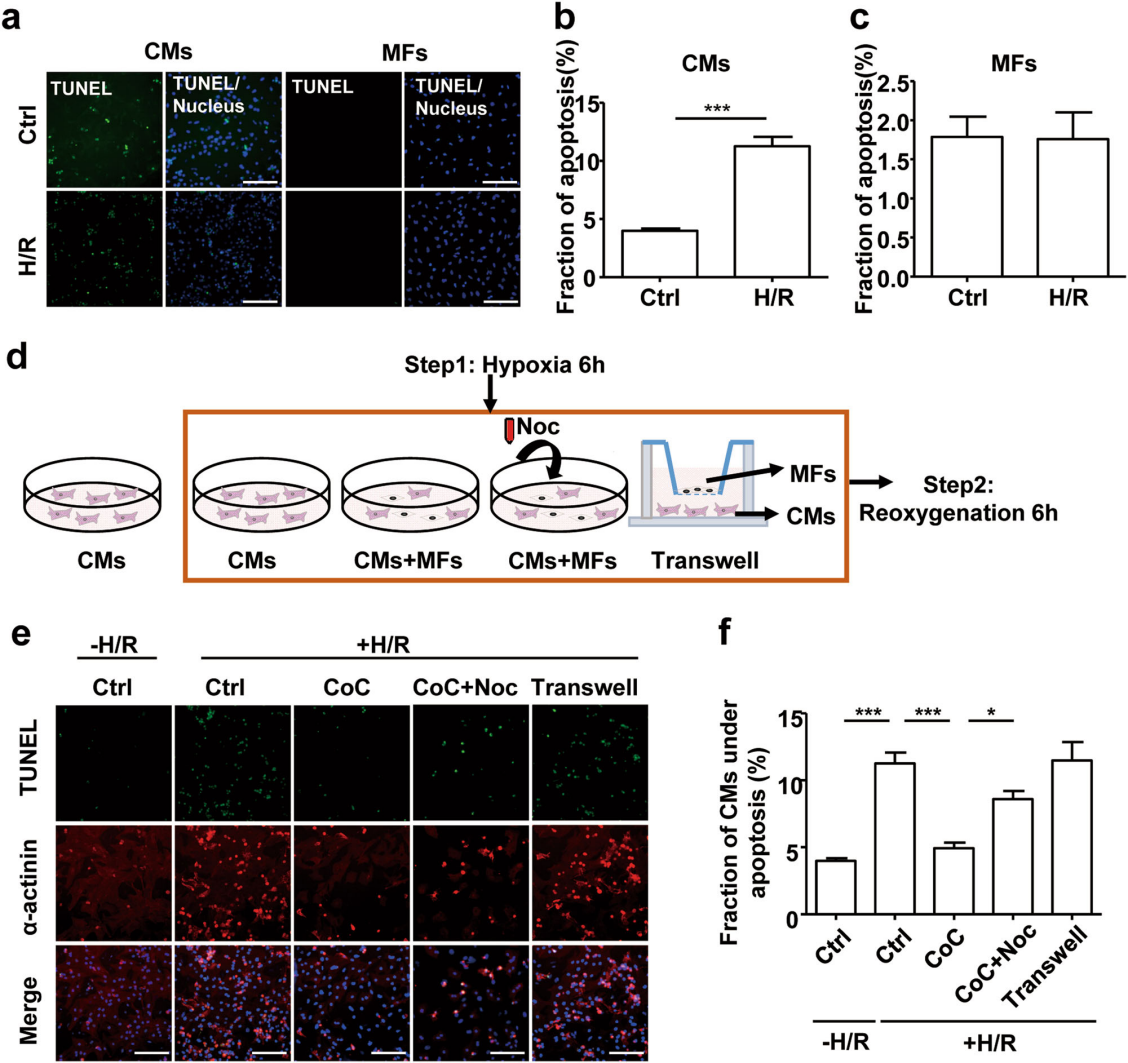
H/R-induced CM apoptosis can be rescued by the MNT-mediated transfer of mitochondria from H/R-treated MFs **a** H/R-induced apoptotic CMs and MFs were identified by TUNEL staining (green) and high-content screening imaging. Scale bar: 100 μm. **b** Quantification of the apoptosis ratio of CMs by high-content screening (*N* = 6). **c** Quantification of the apoptosis ratio of MFs by high-content screening (*N* = 6). **d** Schematic representation of the cell treatments. MFs were cocultured with CMs 16 h prior to the H/R treatment. **e** H/R-induced apoptotic CMs were identified by TUNEL staining (green), and CMs were differentiated by α-actinin staining (red) and high-content screening imaging. Scale bar: 100 μm. **f** Quantification of the apoptosis ratio of CMs by high-content screening (*N* = 6). Data are shown as the mean ± S.E.M. *P < 0. 05 and ***P < 0. 001 using Student’s unpaired two-tailed* t*-test or one-way ANOVA with Tukey’s post hoc test

In vivo, both CMs and MFs may suffer from H/R injury. However, in the present study, in contrast to CMs, MFs did not undergo apoptosis (identified by TUNEL staining) after H/R treatment, suggesting that H/R-exposed MFs may play protective roles similar to those of intact MFs (Fig. 6a–c). To investigate whether H/R-exposed MFs can also rescue distressed CMs, CMs and MFs were cocultured before H/R (Fig. 6d). Using TUNEL staining, we found that apoptosis of distressed CMs increased upon H/R (Fig. 6a, b). However, the increased apoptosis in CMs was blocked when CMs were cocultured with MFs prior to hypoxia exposure (Fig. 6e, f). Rescue from increased apoptosis was not observed under Transwell culture conditions, suggesting that MNTs but not the paracrine pathway were involved in the rescue effects. Nocodazole or colcemid blocked the protective effect of MFs, suggesting that microtubule-mediated mitochondrial transport was involved in rescuing apoptosis (Fig. 6e, f; Supplementary Figure S8d-f). In summary, these results suggest that the apoptosis induced by H/R in CMs can be attenuated by mitochondrial transport within MNTs from H/R-exposed MFs.

## Discussion

**Fig. 7:**
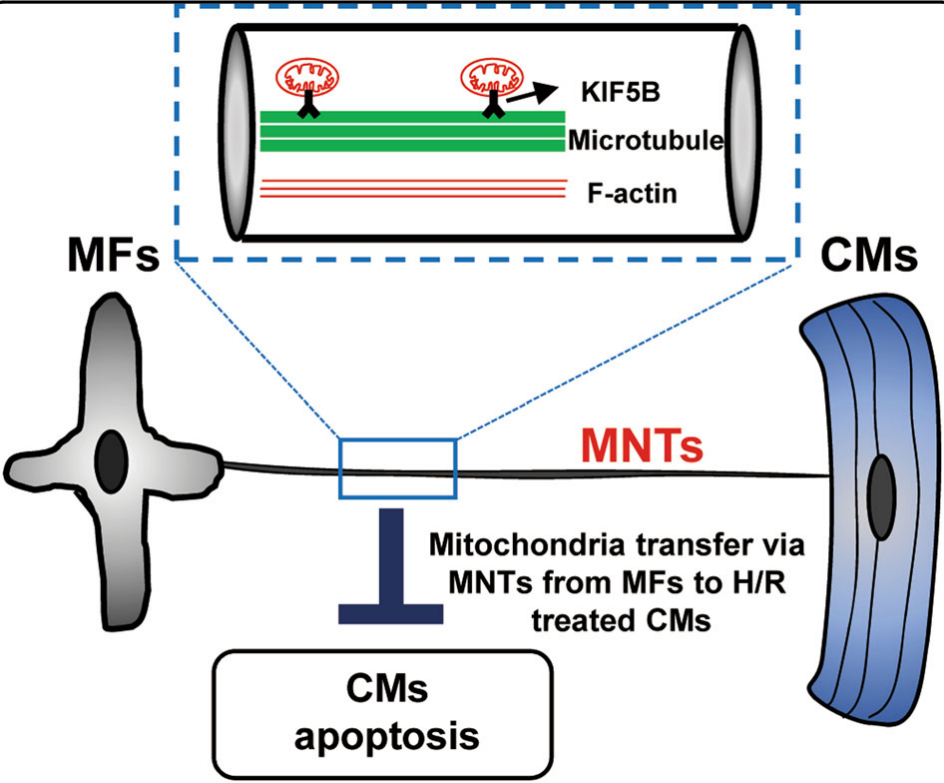
A model of mitochondrial transfer in MNTs between CMs and MFs MNTs physically connect CMs and MFs. Mitochondria are transported along microtubules by the motor protein KIF5B in MNTs and can rescue H/R-induced CM apoptosis. F-actin provides the basic structure of MNTs

In the present study, we demonstrated that mitochondria can be transported in MNTs between CMs and MFs and that the movement of the mitochondria can be characterised as saltatory. Microtubules and the motor protein KIF5B are required for mitochondrial transport in MTNs. Furthermore, we found that intact and hypoxia/reoxygenation-treated MFs could rescue distressed CMs from apoptosis by transferring mitochondria via MNTs (Fig. 7).

Cell-to-cell communication is crucial for multicellular organisms and enables the coordinated interactions of single cells. The types of cell-to-cell communication include indirect paracrine signalling and direct interactions (e.g., gap junction, adhesion junction and MNTs). MNTs are direct highways via which mitochondria can be transferred between cells, thereby allowing for energy and signal exchanges between cells. Previous studies have shown that the transfer of mitochondria between multiple proliferated cell types can occur via MNTs^20,21^. Here, we observed mitochondrial transfer in MNTs between neonatal CMs and MFs. It might be argued that neonatal myocytes undergo division in culture and thus tend to form more MNTs than adult myocytes, which do not divide. Nonetheless, we observed mitochondria within MNTs between isolated adult cardiomyocytes and adult cardiac fibroblasts and also observed mitochondria within MNTs in adult rat heart frozen sections. These results suggested the occurrence of mitochondrial transfer within MNTs in adult rat hearts.

Consistent with previous findings, we observed mitochondria within MNTs between CMs and MFs that displayed vigorous and saltatory movements, similar to the reported transport characteristics of mitochondria in *Drosophila* motor axons^17,22^. In the present study, the mean velocity of the mitochondrial movement in MNTs between CMs and MFs was approximately 17.5 ± 2.1 nm/s, which is similar to that observed between PC12 pheochromocytoma cells^23^ but much lower than that observed between neurons (0.3–1.0 μm/s)^17^. One possible explanation is that the smaller diameter of the MNTs between CMs and MFs may create a higher resistance to mitochondrial movement compared with that between axons.

In multicellular eukaryotes, the movement and distribution of mitochondria depend on the cytoskeleton, which includes actin microfilaments and microtubules. MNTs have been shown to contain microtubules and F-actin^7,24^. Similar to PC12 and myeloid cells^25^, all MNTs between CMs and MFs contain F-actin, whereas only a fraction of MNTs contain microtubules. When the F-actin structure was disrupted, the MNTs appeared to be broken, suggesting that F-actin is required for MNT formation between CMs and MFs. Our study also revealed that microtubules exist in all mitochondria-containing MNTs. We further found that disrupting microtubules disrupted the presence and mobility of mitochondria in MNTs but did not affect the formation of MNTs. Thus, microtubules are involved in the transfer of mitochondria in MNTs. This result is consistent with that of a previous report showing that short-range mitochondrial movement may occur via the actin cytoskeleton, whereas long-distance transportation was microtubule dependent^17^. In addition to microtubules, mitochondrial movement along cytoskeletal elements also relies on the action of molecular motors. We screened the mRNA expression levels of the motor proteins KIF3B, KIF5A, KIF5B and KIF5C, which have been reported to drive the transport of mitochondria^19^. We found that only KIF5B mRNA was highly expressed in CMs and MFs. Other studies have also reported that KIF5B is important for mitochondrial dispersion in the extraembryonic cells of the mouse^26^. Our results demonstrated the existence of KIF5B in MNTs and showed that its depletion inhibited the transfer of mitochondria but did not affect the structure of microtubules, suggesting an important role for KIF5B in mitochondrial transport in MNTs. In summary, KIF5B is an important motor protein involved in mitochondrial transfer in MNTs.

Mitochondria play a key role in maintaining normal function of CMs. Ischaemia, such as that caused by myocardial infarction, can result in mitochondrial damage. The intercellular exchange of mitochondria is a method to restore damaged cells^27^. A previous study has shown that mitochondria can be transferred from MSCs to mtDNA-deficient cells in vitro^28^. Some studies have also unveiled that mitochondrial transfer occurs in vivo, thereby rescuing mitochondrial function^29–31^. Wang and Gerdes^23^ have reported that apoptosis of UV-stressed PC12 cells can be rescued by transferring mitochondria from healthy cells. In an ischaemia model, Cselenyák et al.^32^ used mesenchymal stem cells (MSCs) to rescue cardiomyoblasts from cell death via MNTs. Therefore, the results of previous studies and our study suggest that MSCs and MFs can rescue CMs from apoptosis following ischaemic exposure. Compared with CMs, MFs are relatively more resistant to apoptosis. Therefore, normoxic MFs and H/R-treated MFs can rescue damaged CMs from H/R-induced apoptosis, as shown in the present study (Figs. 5 and 6). Artificial mitochondrial transfer has opened a new field of therapy^33^. Our study demonstrated that resident fibroblasts can also transfer mitochondria via MNTs to play an endogenous protective role in damaged cardiomyocytes under stress. Further study is needed to investigate how to enhance this protective role of MFs under ischaemic stress.

In our previous study, we observed the bidirectional transfer of mitochondria between CMs and MFs under normoxic conditions^7^. However, in the present study, mitochondrial transfer was observed from MFs to hypoxia-treated CMs but not from hypoxia-treated CMs to MFs, suggesting that mitochondrial transfer is unidirectional upon hypoxia exposure, which is consistent with the discovery that in neurons, upon hypoxia exposure, the mitochondrial anterograde (towards the cell periphery) transport velocity decreases, whereas the retrograde (towards the cell body) velocity is enhanced^34^.

Although the present study revealed an important role of MNT-mediated mitochondrial transfer in the protection of distressed CMs by MFs, one limitation must be acknowledged. We demonstrated that MFs can rescue distressed CMs from apoptosis and that this action might be blocked by microtubule depolymerisation reagents nocodazole or colcemid. We hypothesised that mitochondrial transfer was involved in rescuing CMs from apoptosis because mitochondrial transfer is microtubule dependent. However, it is possible that other factors that are dependent on microtubules for transport are also responsible for this rescue effect. Additional work is required to characterise other potential factors.

Together, these results revealed that the motor protein KIF5B is involved in mitochondrial transport in MNTs between CMs and MFs. Moreover, we determined that MNTs contribute to the rescue of CMs from H/R-induced apoptosis by transporting mitochondria. These findings suggest the physiological relevance of MNTs in ischaemic cardiomyopathy and increase our understanding of the function of mitochondrial transfer in MNTs between CMs and MFs.

## Materials and methods

### Primary cell isolation and culture

Neonatal rat CMs and cardiac fibroblasts were isolated and cultured as previously described^35^. Briefly, the hearts of 1-day-old Sprague-Dawley (SD) rats (provided by the Animal Department of Peking University) were quickly excised after deep anaesthetisation with 1.0% isoflurane (CM2L9100, Baxter Healthcare Corporation, New Providence, RI, USA). CMs and cardiac fibroblasts were dispersed by digesting the hearts with 0.1% trypsin (27250-018, Gibco, Carlsbad, CA, USA) and 0.013% collagenase II (17101-015, Gibco) at 37 °C. The isolated cells were plated in 10-cm dishes for 1.5 h to allow the cardiac fibroblasts to attach. The unattached CMs were then transferred to new culture dishes for subsequent treatment. Cardiac fibroblasts and CMs were cultured in Dulbecco’s modified Eagle’s medium (DMEM, 12800-058, Gibco) containing 10% fetal bovine serum and antibiotics (15140-122, Gibco) at 37 °C in 95% O_2_ and 5% CO_2_. First-passage cardiac fibroblasts were used in all experiments and  were identified as MFs (Supplementary Figure S9).

### Mitochondrial tracking

The mitochondria in living cells were transfected for 24 h with Ad-Mito-EGFP, which contained the mitochondria-specific targeting sequence 5′-ATGTCCGTCCTGACGCCGCTGCTGCTGCGGGGCTTGA-3′. Then, cocultured CMs and MFs were treated with microtubule depolymerisation reagents nocodazole (30 μM, 4 h; M1404, Sigma, St. Louis, MO, USA) or colcemid (4 μM, 4 h; 10295892001, Sigma), followed by mitochondrial tracking.

Mitochondrial movement in MNTs between CMs and MFs was tracked in 30 s intervals for 15–20 min using a confocal fluorescence microscope with a 63X/1.4NA oil immersion objective lens (Zeiss Laser Scanning Microscope, LSM780, Zeiss, Oberkochen, Germany). A cell incubation system (Incubator PM S1) was used to ensure that the cells were imaged under 37 °C and 5% CO_2_ conditions. For living cells, we were able to distinguish between CMs and MFs because only CMs can beat. AxioVision 4 Tracking Module (Zeiss) and ImageJ (NIH, Bethesda, MA, USA) were used to track and analyse mitochondrial movement velocity in MNTs.

### Animals and frozen sections of heart tissue

All experimental procedures and animal protocols were approved by the Committee on the Ethics of Animal Experiments (LA2010-039) and conducted in accordance with the Guide for the Care and Use of Laboratory Animals published by the US National Institutes of Health (NIH Publication no. 85–23, revised 2011).

Wild-type male SD rats (10 weeks old, weighing 200–230 g) of specific-pathogen-free grade were provided by the Animal Department of Peking University (Beijing, China). The rats were anaesthetised with 3% isoflurane in oxygen, and pedal pinch reflexes were completely inhibited prior to killing. The heart tissues were then isolated and fixed with optimal cutting temperature compound. Frozen sections of rat hearts (6-μm thick) were obtained using a cryostat (Leica, Wetzlar, Germany), placed on poly-l-lysine-coated glass slides, soaked with acetone, permeabilised with 0.2% Triton X-100 in phosphate-buffered saline (PBS) for 10 min, blocked with 5% bovine serum albumin (BSA) in PBS and then stained with MitoTracker® Green FM (M7514, Invitrogen, Carlsbad, CA, USA). Frozen sections were visualised using a laser scanning confocal microscope with a 63X/1.4NA oil immersion objective lens and an excitation wavelength of 488 nm. All images were acquired with ZEN 2012 software.

### Immunofluorescence

For immunofluorescence staining, the cells were fixed with 4% paraformaldehyde for 15 min in PBS, permeabilised with 0.2% Triton X-100 in PBS, blocked with 5% BSA in PBS and then stained with primary antibodies, α-tubulin (ab52866, Abcam, Cambridge, MA, USA), α-actinin (A7811, Santa Cruz Biotech, CA, USA) or KIF5B (ab167429, Abcam), followed by Alexa Fluor 546/633 secondary antibodies. Rhodamine phalloidin dye (R415, Invitrogen) and WGA Alexa Fluor® 488 conjugate (WGA, W11261, Invitrogen) were incubated with the cells for 20 min at room temperature for F-actin or cell membrane staining. To detect the mitochondrial transfer shown in Fig. 5a, MitoTracker® Orange CMTMRos (Mito Orange, M7510 Invitrogen) was incubated with the cells for 20 min at 37 °C.

Stained cells were visualised and analysed using a laser scanning confocal microscope with a 63X/1.4NA oil immersion objective lens and excitation wavelengths of 488, 549 and 633 nm. All images were acquired using ZEN 2012 software.

### Lentiviral infection

Small interfering RNA was used to knock down KIF5B expression in cells. CMs and MFs were infected by the lentiviral vector LV9-pGLV-u6-RFP carrying KIF5B siRNA1 or KIF5B siRNA2 (targeting the 1395–1416 nt region relative to the first nucleotide of the start codon, 5′-GGATCAAGATAATATGCAAGC-3′ or 5′-GCAGCTGAAATGATGGCATCA-3′) or a scramble siRNA (5′-TTCTCCGAACGTGTCACGTTTC-3′) for 24 h at a multiplicity of infection of 100 with 5 mg/ml polybrene.

### Hypoxia/reoxygenation (H/R)

CMs were seeded on a 24-well plate at a density of 4 × 10^4^ cells per well and cultured at 37 °C for 2 days. The medium was replaced with serum-free DMEM prior to culturing in hypoxic buffer containing 118 mM NaCl, 24 mM NaHCO_3_, 1.0 mM NaH_2_PO_4_, 1.2 mM MgCl_2_, 2.5 mM CaCl_2_-2H_2_O, 16 mM KCl, 20 mM sodium lactate and 10 mM 2-deoxyglucose (pH 6.2), as described previously^36^. The cells were incubated at 37 °C in an atmosphere of 5% CO_2_ and adjusted to 1% O_2_ during the hypoxic stress period. For reoxygenation, the hypoxia buffer was replaced with normal medium, and the cells were incubated under normoxic incubator conditions. During the reoxygenation period, an equivalent number of MFs were added directly to the CMs or to a Transwell plate, with a 0.3 µm polyester membrane (3460, Costar Corning, NY, USA) placed above the cultured CMs, which constituted the coculture and Transwell culture systems, respectively.

### Apoptosis detection

CM apoptosis was determined by TUNEL assay (G3250, Promega Corporation, Fitchburg, WI, USA). CMs were identified by α-actinin staining. The cell nuclei were stained with 4',6-diamidino-2-phenylindole (62247, Invitrogen) or Hoechst 33342 (H3570, Invitrogen). TUNEL-positive cells were quantified by high-content screening (Cellomics ArrayScan Infinity, Thermo Fisher Scientific, Rockford, IL, USA) in 20 randomly chosen sites in every well of the cell culture plate. The percentage of apoptotic CMs was expressed as the apoptotic index, which was calculated using the following equation: apoptosis index (%) = (apoptotic CM nuclei count / total CM nuclei count) × 100%.

### Statistical analysis

Data are represented as the mean ± S.E.M. of at least three independent experiments. Statistical analyses were performed using GraphPad Prism 5.0 (GraphPad Software Inc., La Jolla, CA, USA). For parametric data, Student’s *t*-test or analysis of variance (ANOVA) combined with Tukey’s post hoc test was used to analyse the differences among groups if the data were normally distributed. For data with unequal variances, Welch’s *t*-test was used. For non-parametric data, the Mann–Whitney *U*-test with the exact method was used to analyse the differences between two groups. The Kruskal–Wallis ANOVA combined with post hoc Dunn’s multiple comparison test was performed when more than two groups were evaluated. Differences were considered statistically significant at *P < *0.05.

## Electronic supplementary material

supplemental data

movie 1

movie 2

movie 3

movie 4

movie 5

movie 6

movie 7

## References

[1] Baudino TA (2008). Cell patterning: interaction of cardiac myocytes and fibroblasts in three-dimensional culture. Microsc. Microanal..

[2] Porter KE, Turner NA (2009). Cardiac fibroblasts: at the heart of myocardial remodeling. Pharmacol. Ther..

[3] Zhang J, Zhang Y (2013). Membrane nanotubes: novel communication between distant cells. Sci. China Life Sci..

[4] Burtey A (2015). Intercellular transfer of transferrin receptor by a contact-, Rab8-dependent mechanism involving tunneling nanotubes. FASEB J..

[5] Rustom A, Saffrich R, Markovic I, Walther P, Gerdes HH (2004). Nanotubular highways for intercellular organelle transport. Science.

[6] He K (2010). Intercellular transportation of quantum dots mediated by membrane nanotubes. ACS Nano.

[7] He K (2011). Long-distance intercellular connectivity between cardiomyocytes and cardiofibroblasts mediated by membrane nanotubes. Cardiovasc. Res..

[8] Dixon IM, Davies JJ (2011). Fibroblasts are coupled to myocytes in heart muscle by nanotubes: a bigger and better syncytium?. Cardiovasc. Res..

[9] Wang C, Youle RJ (2009). The role of mitochondria in apoptosis*. Annu. Rev. Genet..

[10] Kale J, Liu Q, Leber B, Andrews DW (2012). Shedding light on apoptosis at subcellular membranes. Cell.

[11] Yaniv Y, Juhaszova M, Sollott SJ (2013). Age-related changes of myocardial ATP supply and demand mechanisms. Trends Endocrinol. Metab..

[12] Forini F, Nicolini G, Iervasi G (2015). Mitochondria as key targets of cardioprotection in cardiac ischemic disease: role of thyroid hormone triiodothyronine. Int J. Mol. Sci..

[13] Liu K (2014). Mesenchymal stem cells rescue injured endothelial cells in an in vitro ischemia-reperfusion model via tunneling nanotube like structure-mediated mitochondrial transfer. Microvasc. Res..

[14] Islam MN (2012). Mitochondrial transfer from bone-marrow-derived stromal cells to pulmonary alveoli protects against acute lung injury. Nat. Med..

[15] Long X (1997). p53 and the hypoxia-induced apoptosis of cultured neonatal rat cardiac myocytes. J. Clin. Invest..

[16] Morris RL, Hollenbeck PJ (1995). Axonal transport of mitochondria along microtubules and F-actin in living vertebrate neurons. J. Cell Biol..

[17] MacAskill AF, Kittler JT (2010). Control of mitochondrial transport and localization in neurons. Trends Cell Biol..

[18] Vale RD (2003). The molecular motor toolbox for intracellular transport. Cell.

[19] Hirokawa N, Noda Y (2008). Intracellular transport and kinesin superfamily proteins, KIFs: structure, function, and dynamics. Physiol. Rev..

[20] Vallabhaneni KC, Haller H, Dumler I (2012). Vascular smooth muscle cells initiate proliferation of mesenchymal stem cells by mitochondrial transfer via tunneling nanotubes. Stem Cells Dev..

[21] Pasquier J (2013). Preferential transfer of mitochondria from endothelial to cancer cells through tunneling nanotubes modulates chemoresistance. J. Transl. Med..

[22] Pilling AD, Horiuchi D, Lively CM, Saxton WM (2006). Kinesin-1 and Dynein are the primary motors for fast transport of mitochondria in Drosophila motor axons. Mol. Biol. Cell.

[23] Wang X, Gerdes HH (2015). Transfer of mitochondria via tunneling nanotubes rescues apoptotic PC12 cells. Cell Death Differ..

[24] Chauveau A, Aucher A, Eissmann P, Vivier E, Davis DM (2010). Membrane nanotubes facilitate long-distance interactions between natural killer cells and target cells. Proc. Natl. Acad. Sci. USA.

[25] Davis DM, Sowinski S (2008). Membrane nanotubes: dynamic long-distance connections between animal cells. Nat. Rev. Mol. Cell Biol..

[26] Tanaka Y (1998). Targeted disruption of mouse conventional kinesin heavy chain, kif5B, results in abnormal perinuclear clustering of mitochondria. Cell.

[27] Spees JL, Olson SD, Whitney MJ, Prockop DJ (2006). Mitochondrial transfer between cells can rescue aerobic respiration. Proc. Natl. Acad. Sci. USA.

[28] Cho YM (2012). Mesenchymal stem cells transfer mitochondria to the cells with virtually no mitochondrial function but not with pathogenic mtDNA mutations. PLoS ONE.

[29] Tan AS (2015). Mitochondrial genome acquisition restores respiratory function and tumorigenic potential of cancer cells without mitochondrial DNA. Cell Metab..

[30] Dong LF (2017). Horizontal transfer of whole mitochondria restores tumorigenic potential in mitochondrial DNA-deficient cancer cells. Elife.

[31] Osswald M (2015). Brain tumour cells interconnect to a functional and resistant network. Nature.

[32] Cselenyak A, Pankotai E, Horvath EM, Kiss L, Lacza Z (2010). Mesenchymal stem cells rescue cardiomyoblasts from cell death in an in vitro ischemia model via direct cell-to-cell connections. BMC Cell Biol..

[33] Caicedo A, Aponte PM, Cabrera F, Hidalgo C, Khoury M (2017). Artificial mitochondria transfer: current challenges, advances, and future applications. Stem Cells Int.

[34] Li Y (2009). HUMMR, a hypoxia- and HIF-1alpha-inducible protein, alters mitochondrial distribution and transport. J. Cell Biol..

[35] Liao W, Wang S, Han C, Zhang Y (2005). 14-3-3 proteins regulate glycogen synthase 3beta phosphorylation and inhibit cardiomyocyte hypertrophy. FEBS J..

[36] Das A, Smolenski A, Lohmann SM, Kukreja RC (2006). Cyclic GMP-dependent protein kinase Ialpha attenuates necrosis and apoptosis following ischemia/reoxygenation in adult cardiomyocyte. J. Biol. Chem..

