# Supplementary material for: Mitochondria are transported along microtubules in membrane nanotubes to rescue distressed cardiomyocytes from apoptosis
Source: Cell Death Dis. 2018 Jan 23;9(2):81. doi: 10.1038/s41419-017-0145-x (PMC5833423; doi:10.1038/s41419-017-0145-x)

**Mitochondria are transported along microtubules in membrane nanotubes to rescue distressed cardiomyocytes from apoptosis**

Jing Shen*, Jiang-Hui Zhang*, Han Xiao*, Ji-Min Wu, Kang-Min He, Zhi-Zhen Lv, Zi-Jian Li, Ming Xu, You-Yi Zhang

Department of Cardiology and Institute of Vascular Medicine, Peking University Third Hospital; Key Laboratory of Cardiovascular Molecular Biology and Regulatory Peptides, Ministry of Health; Key Laboratory of Molecular Cardiovascular Science, Ministry of Education; Beijing Key Laboratory of Cardiovascular Receptors Research. Beijing 100191, China

* These authors contributed equally to this work.

**Corresponding author**

You-Yi Zhang, MD, PhD

Institute of Vascular Medicine

Peking University Third Hospital

No .49, Huayuan Bei Road, Haidian District

Beijing, 100191, China

Tel.: + 86 -10- 82802306

Fax: + 86 -10- 62361450

Email: zhangyy@bjmu.edu.cn

**SUPPLEMENTAL MATERIALS**

**Supplemental Methods**

***Isolation and culture of cardiomyocytes and cardiac fibroblasts from adult rats***

Wild-type male SD rats (10 weeks old, weighing 200-230 g) of specific-pathogen-free (SPF) grade were provided by the Animal Department of Peking University (Beijing, China). The rats were anaesthetised with 3% isoflurane in oxygen; then, the hearts were isolated and perfused with buffer A (NaCl 137 mM; HEPES 20 mM; D-Glucose 10 mM; KCl 5.4 mM; MgCl2.6H2O 1.2 mM; Na2PO4.2H2O; taurine 10 mM) for 8-10 min, followed by enzyme solution (buffer A with 0.667 mg/ml collagenase II 1 mg/ml Bovine serum albumin and 0.05 mM CaCl2) for 20-30 min, and then minced into small pieces and resuspended. Calcium reintroduction was performed by gradually increasing the calcium concentration at intervals of 10-15 min. Adult cardiomyocytes were plated on laminin-coated plates.

SD rats were anaesthetised, and ventricles were minced and digested with collagenase Ⅱ (330 u/ml) and then placed in plates. After two hours, the plates were washed with PBS; then, cardiac fibroblasts were cultured in DMEM containing 10% foetal bovine serum.

***Quantitative real-time polymerase chain reaction (PCR)***

Total RNA was extracted from cultured cardiomyocytes (CMs), myofibroblasts (MFs) or neonatal rat brain tissue with TRIzol Reagent (15596018, Life Technologies, Gaithersburg, MD, USA), and 1 μg of RNA was used for reverse transcription. The expression of kinesin mRNA was quantified by real-time PCR with SYBR Green in a Mastercycler ep realplex System (Eppendorf, Hamburg, Germany). The amplification procedure was performed at 94 °C for 2 min, followed by 40 cycles of 94 °C for 30 s, 55 °C for 15 s and 72 °C for 30 s. The primers sequences are provided below:

***Primer sequences***

|  | **Forward Sequence** | **Reverse Sequence** |
| --- | --- | --- |
| **KIF3B** | ATCATACAAACGAGCAGCAG | GTCTCTTTCAGTTCCAAGGTC |
| **KIF5A** | TTTTCCCGAGTGTATCAGCTAC | TTCTGCCGTCTCTTGGTGGAG |
| **KIF5B** | GGGAATAAGACTCTACGGAAC | GGCAGCATCTGTAAAACTACC |
| **KIF5C** | CAGATTGAAAACGAGGCGGC | AAGTCCAGCCCCCTTAACAC |
| **GAPDH** | ATCAAGAAGGTGGTGAAGCA | AAGGTGGAAGAATGGGAGTTG |

***Western blot analysis***

The cells were lysed, and the BCA Protein Assay kit (23228; 23224, Thermo Fisher Scientific) was used to measure protein concentrations. Samples containing 50 μg of protein were electrophoresed by 10% sodium dodecyl sulphate polyacrylamide gel electrophoresis (SDS-PAGE) and transferred to nitrocellulose membranes. After blocking with 5% milk in phosphate-buffered saline (PBS), the membranes were incubated with the appropriate primary antibodies KIF5B (ab167429, Abcam) or eIF5B (#sc-282, Santa Cruz Biotechnology, Santa Cruz, CA, USA) or αSMA (ab32575, Abcam) or fibronectin (ab2413, Abcam) overnight at 4 °C, followed by secondary antibodies for 1 h at room temperature. Immunostained bands were visualised using the Pierce ® ECL Western Blotting Substrate (WBKLS0500, Millipore, Billerica, MA, USA).

**Supplemental figures and figure legends**

**
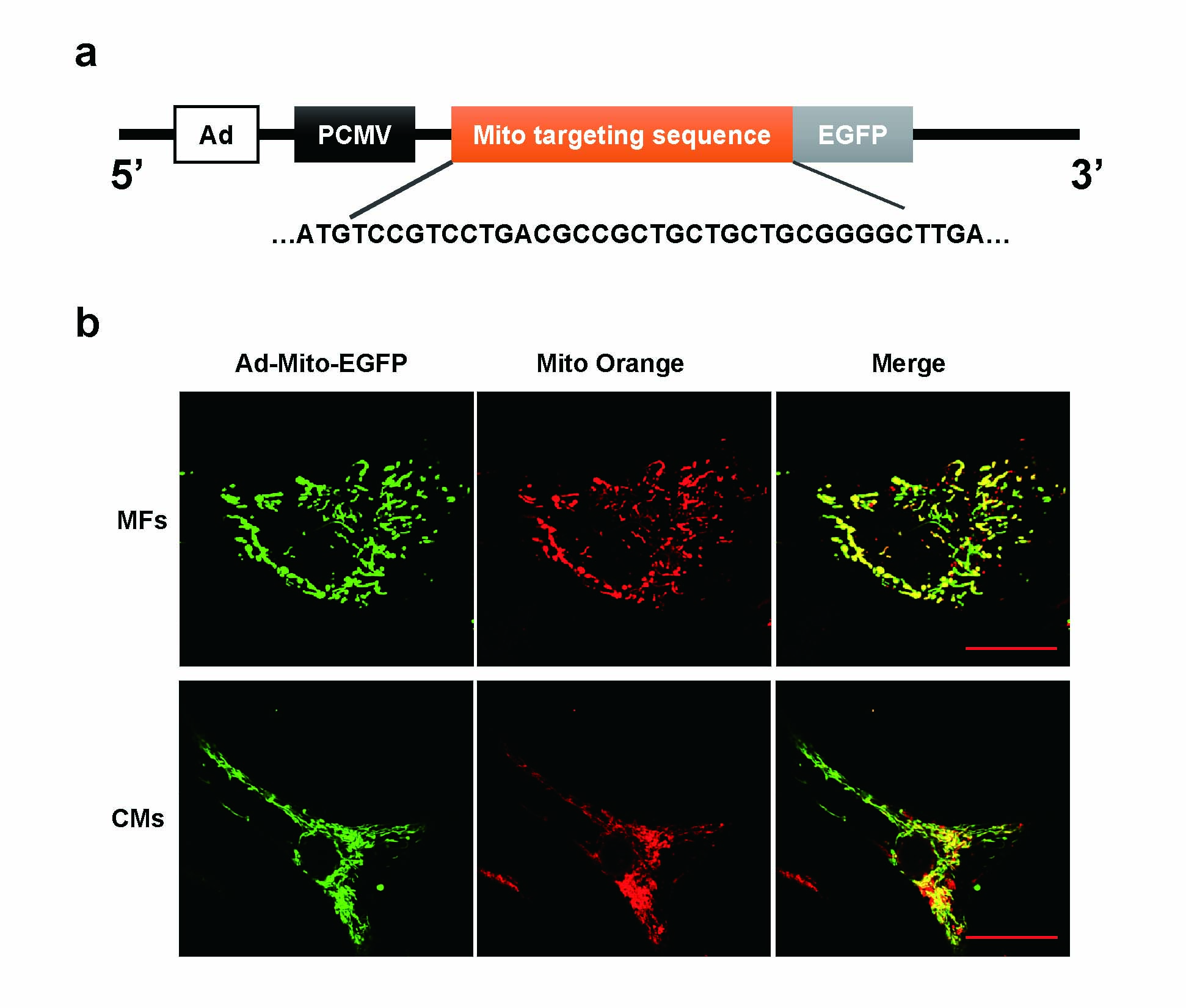
**

**Figure S1.** Mitochondria in neonatal rat ventricular cardiomyocytes (CMs) and cardiac fibroblasts (MFs) were specifically labelled by adenovirus-mitochondria-enhanced green fluorescent protein (EGFP) (Ad-Mito-EGFP). (a) The construct and mitochondria-specific targeting sequence of Ad-Mito-EGFP. (b) Mitochondria in Ad-Mito-EGFP-infected CMs and MFs were stained with Mito Orange (red). Confocal micrographs show the co-localisation of the green and red signals. Scale bar: 25 μm.

**
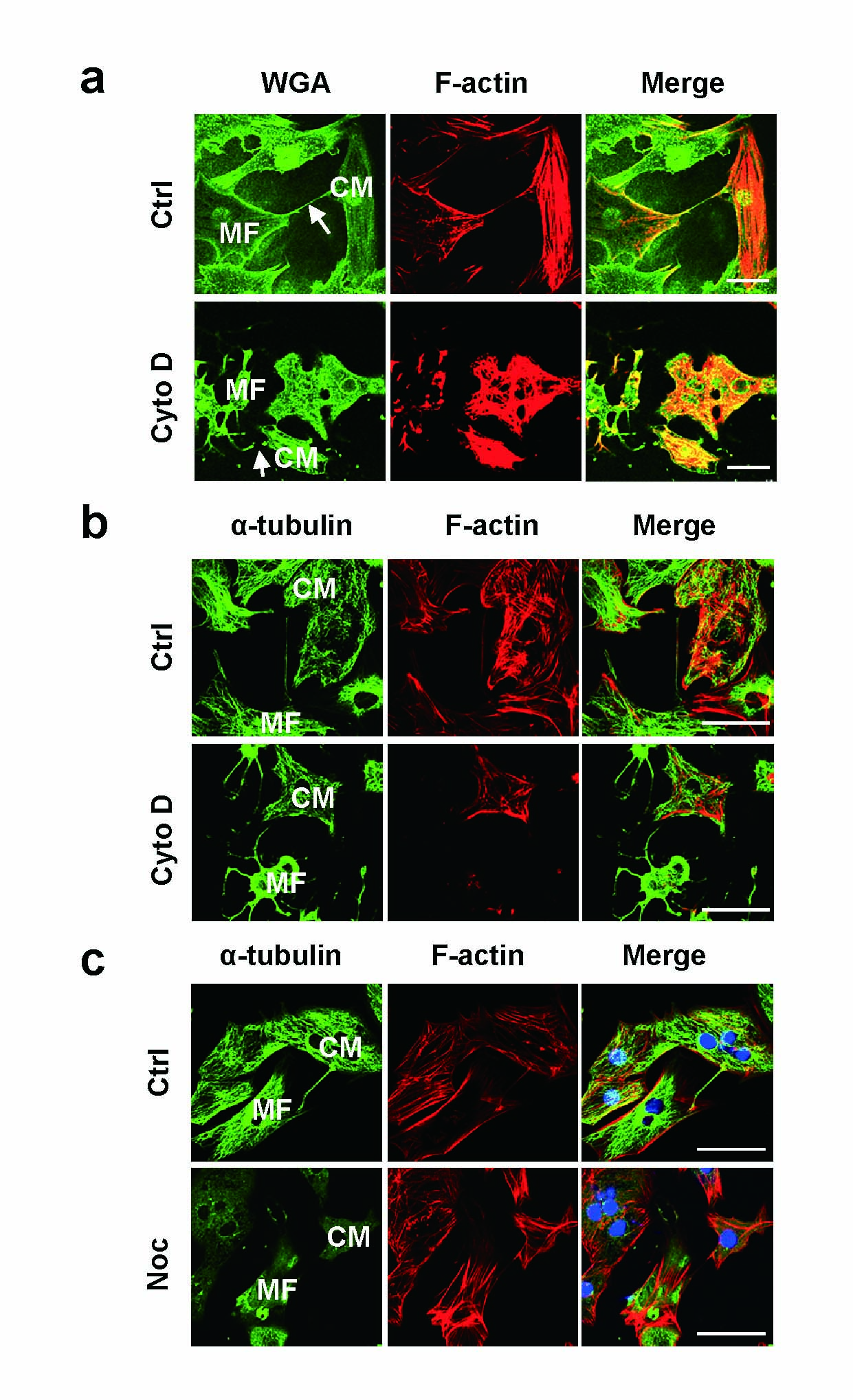
**

**Figure S2.** F-actin is essential for membrane nanotubes (MNTs). CMs and MFs were cocultured and treated with or without cytochalasin D (Cyto D) or nocodazole (Noc). (a) The cell membrane was labelled with the membrane dye WGA Alexa Fluor 488 conjugate (WGA, green), and F-actin was labelled with rhodamine phalloidin (red). White arrows show the intact and broken MNTs. (b) After being treated with Cyto D, cells were dual-labelled for microtubules and F-actin with α-tubulin antibodies (green) and rhodamine phalloidin (red), respectively. (c) After treatment with Noc, cells were dual-labelled for microtubules and F-actin with α-tubulin antibodies and rhodamine phalloidin (red), respectively. Scale bar: 25 μm.

**
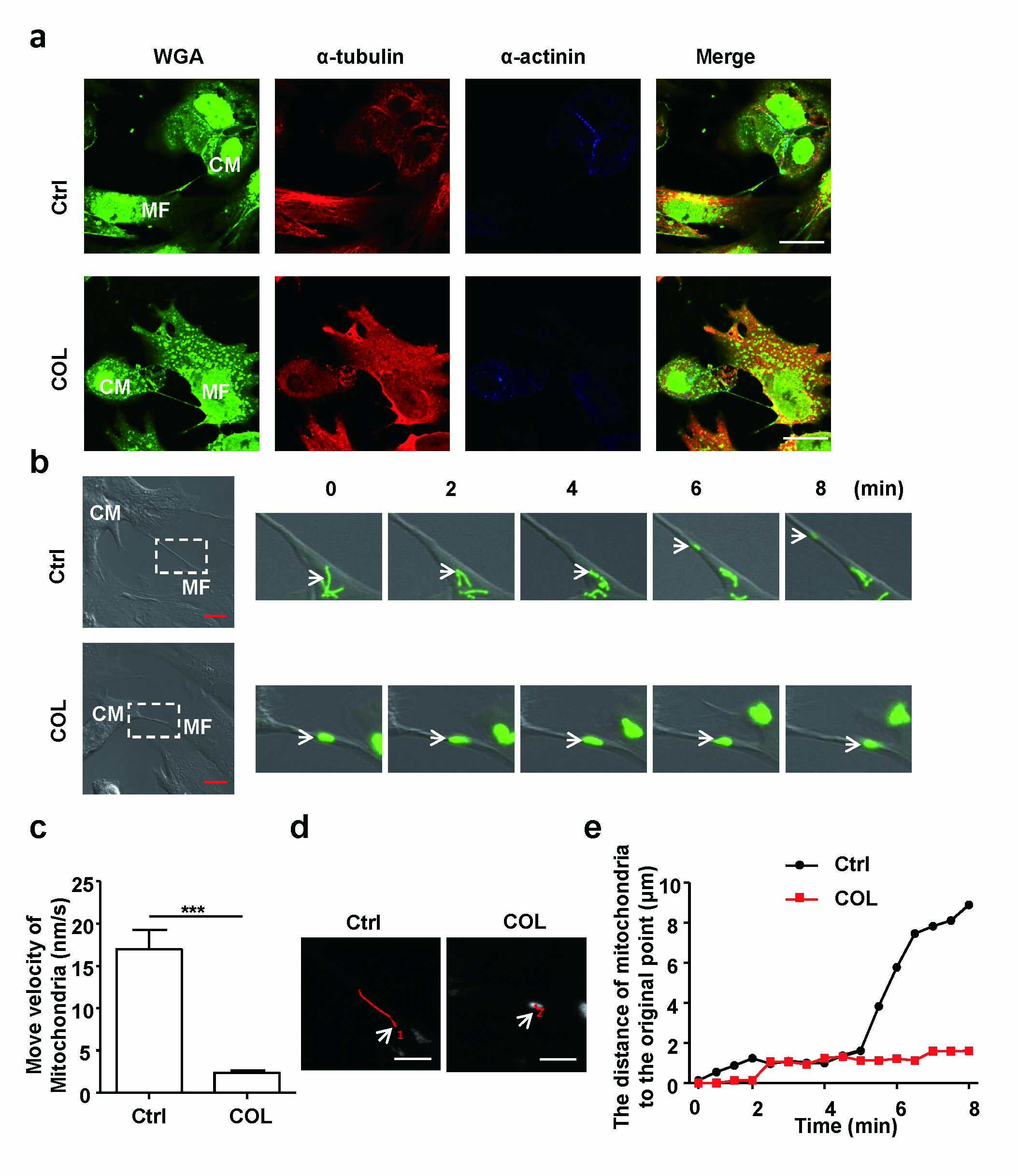
**

**Figure S3.** The mitochondrial transfer in MNTs between CMs and MFs was dependent on microtubules. (a) Cocultured CMs and MFs were triple labelled by WGA (green), α-tubulin (red) and α-actinin (blue) and then treated with or without the microtubule depolymerisation reagent colcemid (COL). WGA-labelled MNTs were still detectable in cells treated with colcemid. Scale bar: 20 μm. (b) A series of confocal images from time-lapse movies show the movement of Ad-Mito-EGFP-labelled mitochondria (arrows) in MNTs between CMs and MFs, with or without colcemid treatment. Scale bar: 10 μm. (c) Mean movement velocities of mitochondria in MNTs between CMs and MFs, with or without colcemid treatment (n = 10 mitochondria from 3 independent experiments). (d) The mobile trajectories of mitochondria in MNTs (in panel a) were shorter after colcemid treatment. The trajectories are represented as red lines, and the arrows point to the starting points. Scale bar: 5 μm. (e) Distance changes over time (relative to the starting point) of the mitochondria in panel b. Data are shown as the mean ± standard error of the mean (S.E.M). *** P < 0.001 using Welch’s t-test.


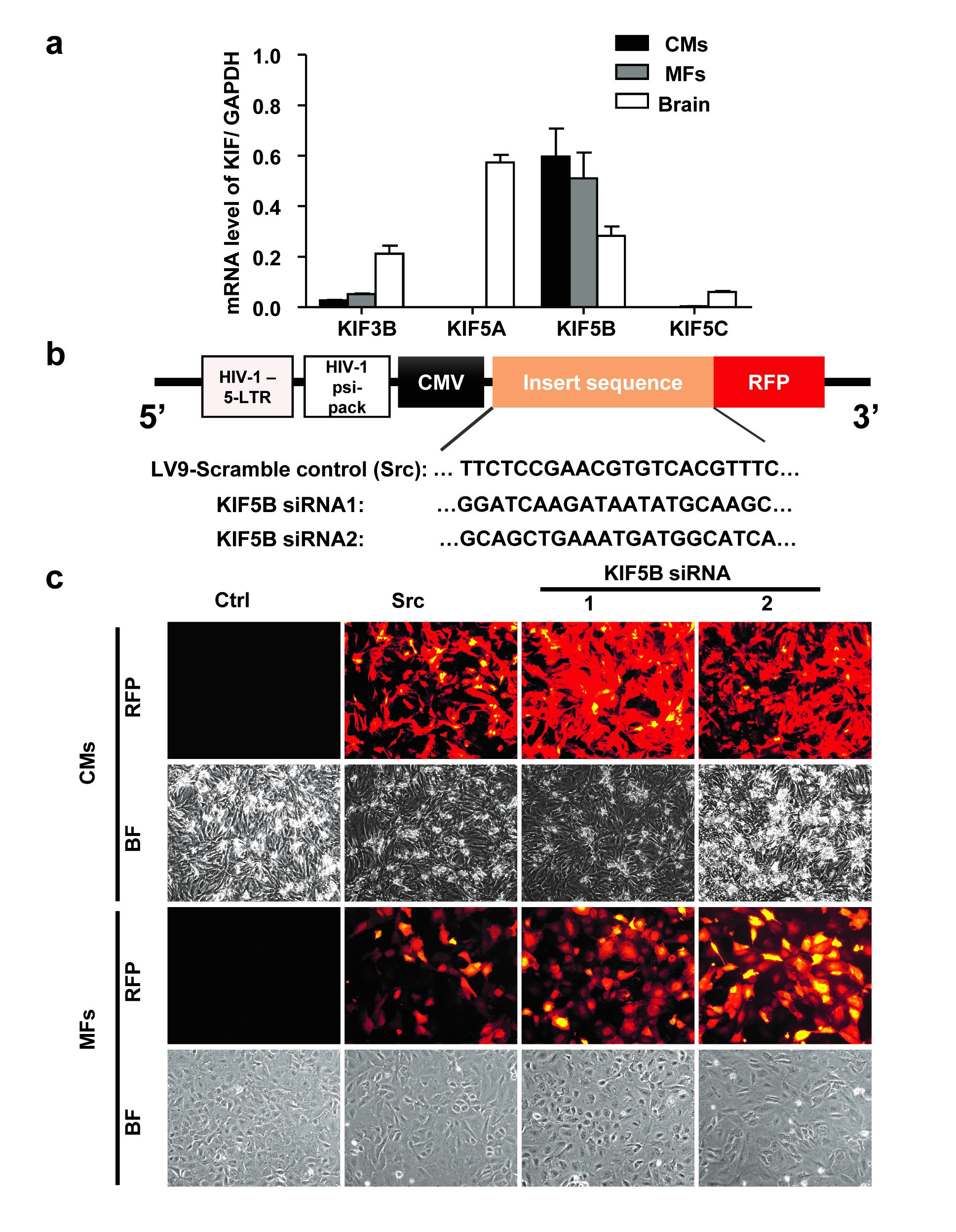


**Figure S4.** Structure and transfection efficiency of lentiviruses containing kinesin family member 5B (KIF5B) siRNA. (a) The mRNA levels of KIF3B, KIF5A, KIF5B and KIF5C in the CMs and MFs were analysed using real-time PCR. Brain tissue was used as a positive control (N = 3). (b) The structure and insert sequence of KIF5B siRNA. (c) CMs and MFs were cultured in medium containing the lentivirus under a multiplicity of infection (MOI) of 100, with 5 mg/ml polybrene for 5 d. Most cells were successfully infected with the lentivirus (red).


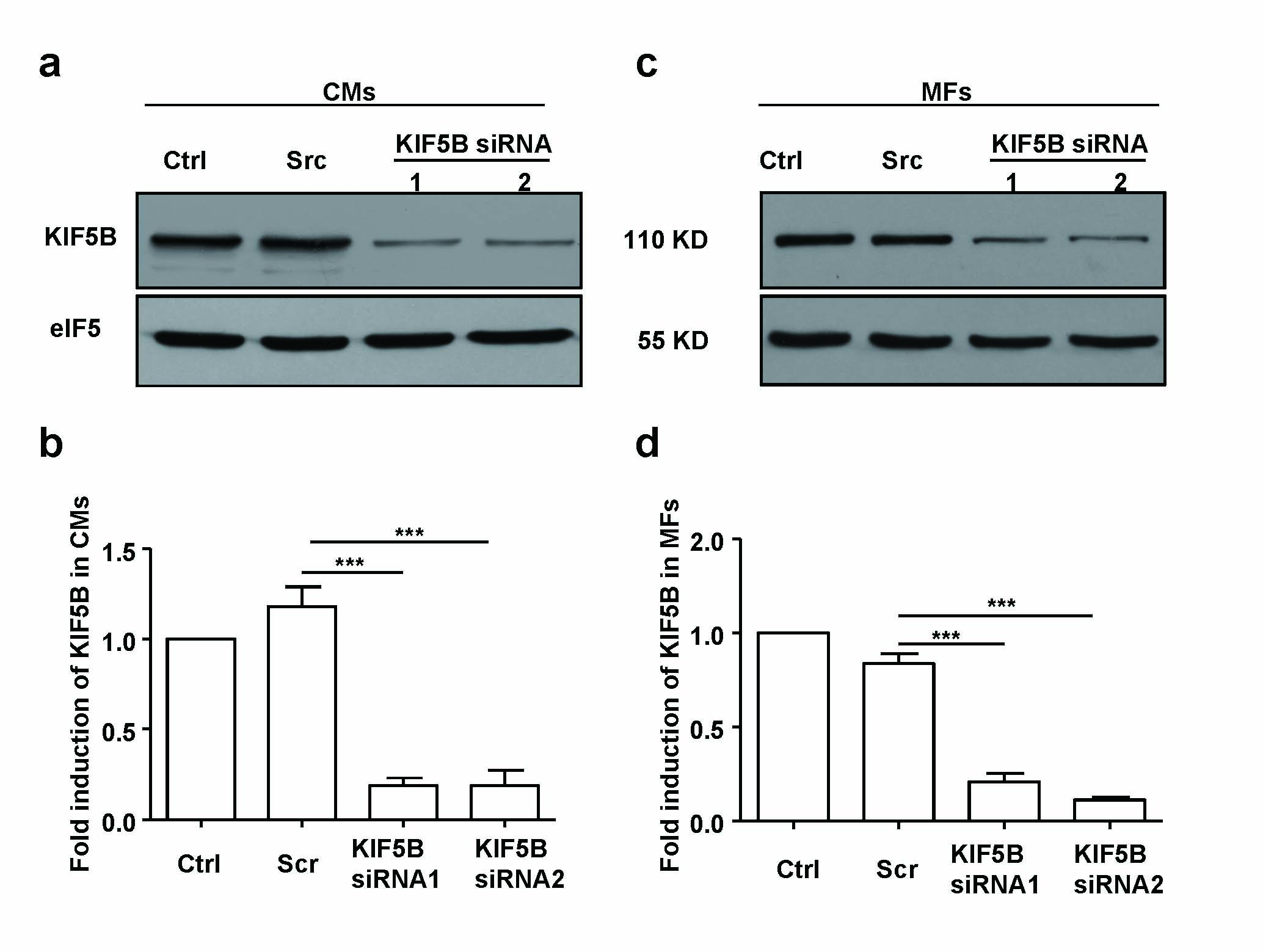


**Figure S5.** The expression of KIF5B was effectively depleted by KIF5B siRNA. (a) The expression of KIF5B in CMs treated with scramble siRNA (Scr) or KIF5B siRNA was determined *via* western blot analysis (KIF5B: 110 kDa; eukaryotic translation initiation factor 5 [eIF5]: 55 kDa). (b) Quantification of KIF5B expression by western blot analysis in CMs. (c) The expression of KIF5B in the MFs treated with Scr or KIF5B siRNA, as determined *via* western blot analysis. (d) Quantification of KIF5B expression in MFs by western blot analysis (N = 3). Data are presented as the mean ± S.E.M. *** *P* < 0.001 using one-way ANOVA with Tukey’s post hoc test.


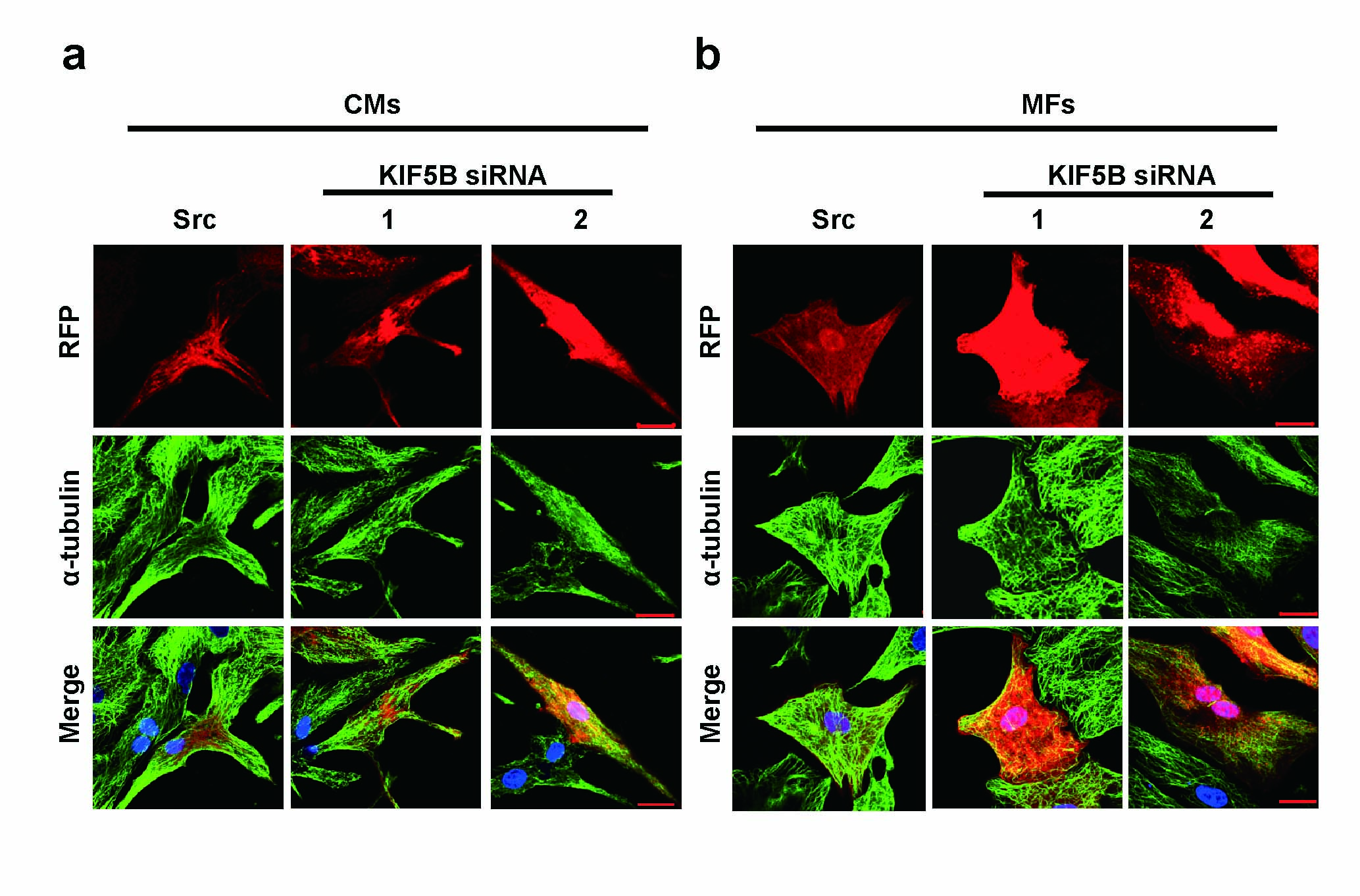


**Figure S6.** KIF5B depletion in CMs and MFs did not affect microtubule structure. (a) CMs infected with scramble siRNA (Scr) or KIF5B siRNA 1/2 (red) were stained with α-tubulin antibodies (green) to label the microtubules and with Hoechst 33342 dye (blue) to label the nuclei. (b) MFs infected with Scr or KIF5B siRNA 1/2 (red) were stained with α-tubulin antibodies (green) and Hoechst 33342 dye (blue). The images were obtained by confocal microscopy. Scale bar: 20 μm.


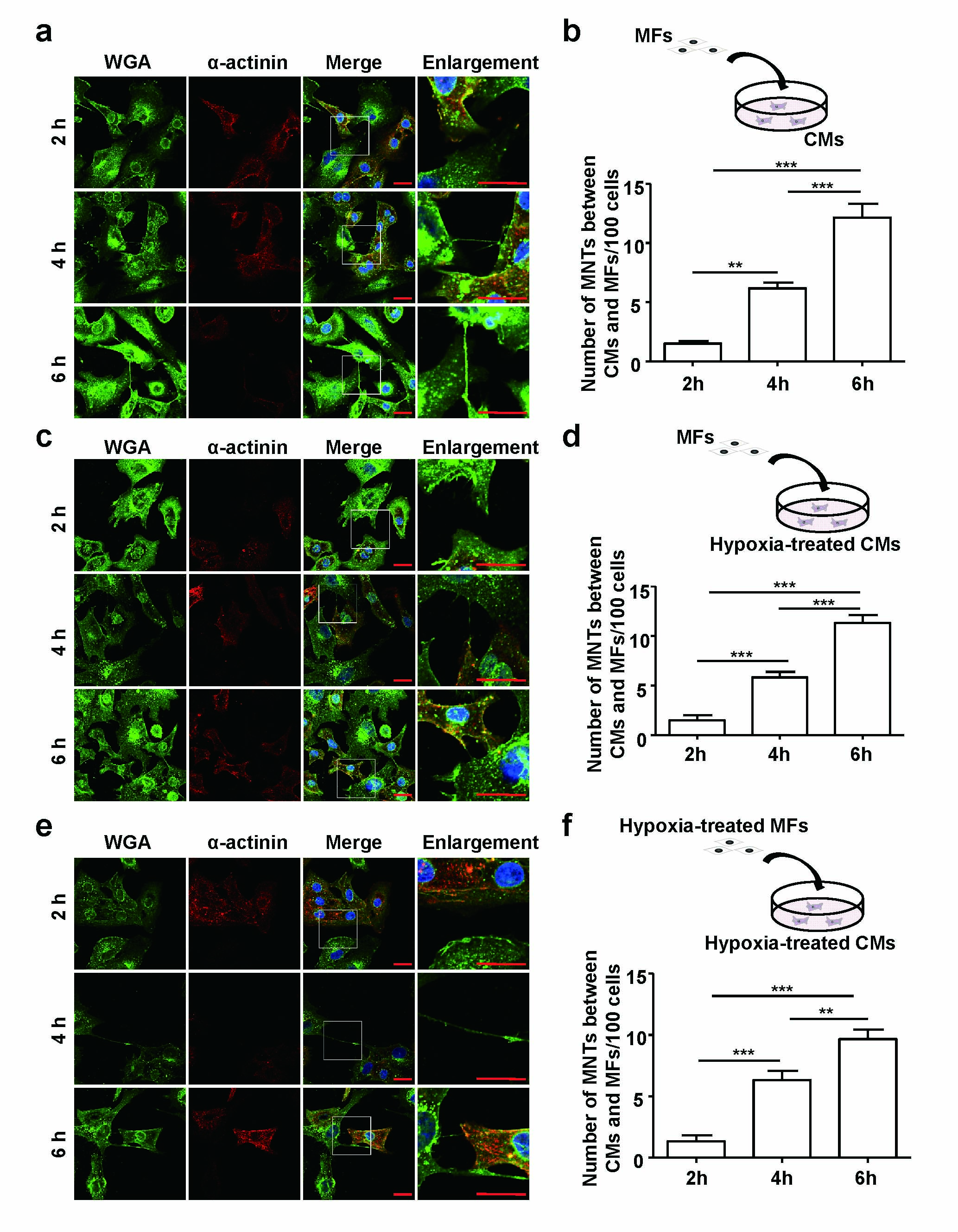


**Figure S7.** The number of MNTs formed between CMs and MFs at different time points post-coculture. (a) Representative images of MNTs between CMs and MFs at the indicated time points after coculture. Cocultured CMs and MFs were labelled with the green membrane dye WGA, CM-specific sarcomeric α-actinin (red) and the nuclei dye Hoechst 33342 (blue). The white box region is enlarged and shown on the right. (b) Quantification of MNT numbers between CMs and MFs (calculated in 100 cells). (c) Representative images of MNTs between hypoxia-treated CMs and intact MFs at the indicated time points after coculture, and (d) quantification of MNTs. (e) Representative images of MNTs between hypoxia-treated CMs and hypoxia-treated MFs at the indicated time points after coculture, and (f) quantification of MNTs (N = 6 for each experiment). Scale bar: 20 μm. Data are presented as the mean ± S.E.M. ** *P* < 0.01 and *** *P* < 0.001 using one-way ANOVA with Tukey’s post hoc test.

**
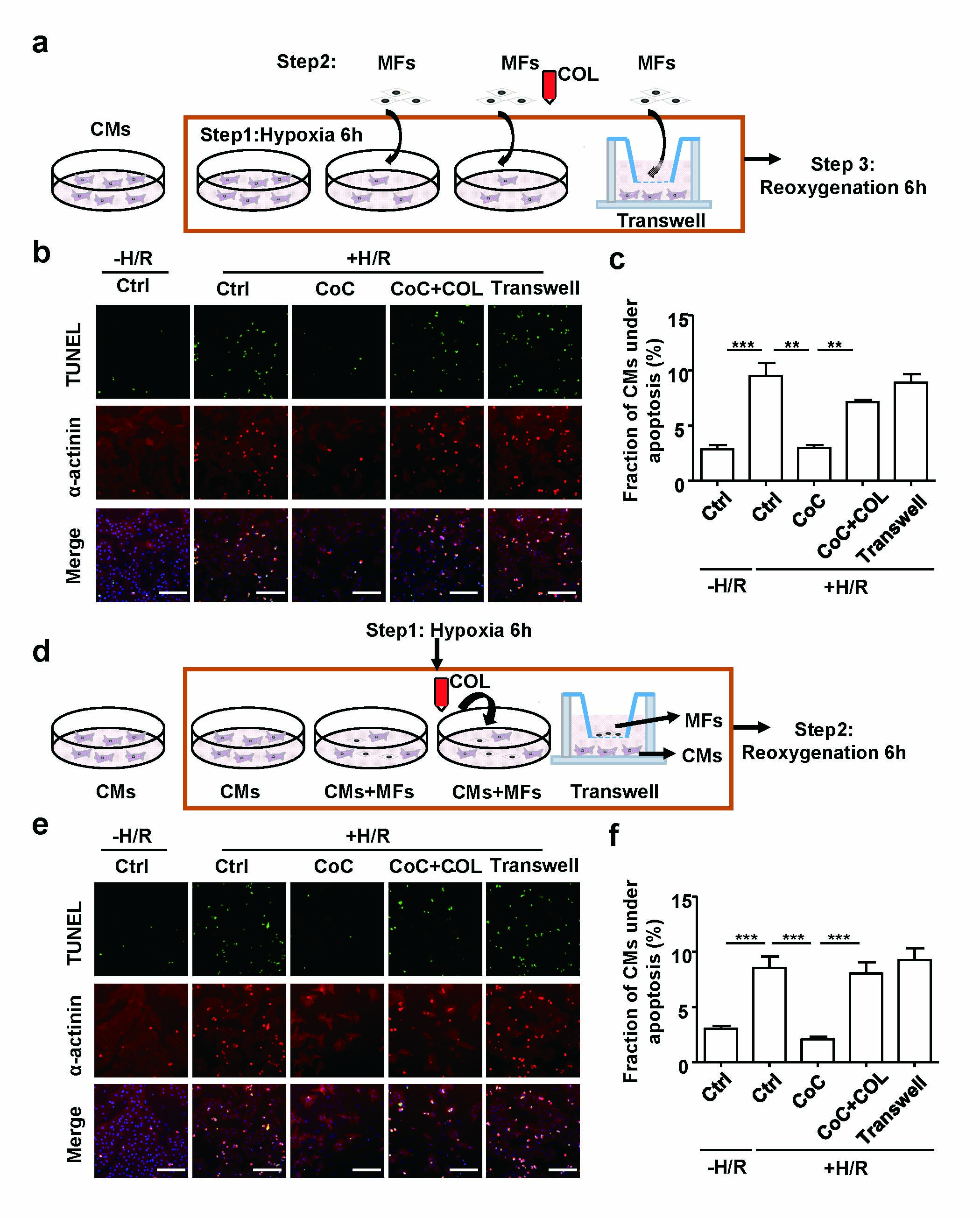
**

**Figure S8.** H/R-induced CM apoptosis can be rescued by the mitochondrial transfer in MNTs from MFs to CMs. (a) & (d) Schematic representation of the cell treatments. (b) & (e) H/R-induced CM apoptosis was identified by TUNEL staining (green) and high-content screening imaging. CMs were differentiated by α-actinin staining (red). CoC, coculture. COL, colcemid. Scale bar: 100 μm. (c) & (f) Quantification of the fraction of apoptotic CMs by high-content screening (N = 5). Data are shown as the mean ± S.E.M. ** *P* < 0. 01 and *** *P* < 0. 001 using one-way ANOVA with Tukey’s post hoc test.

**
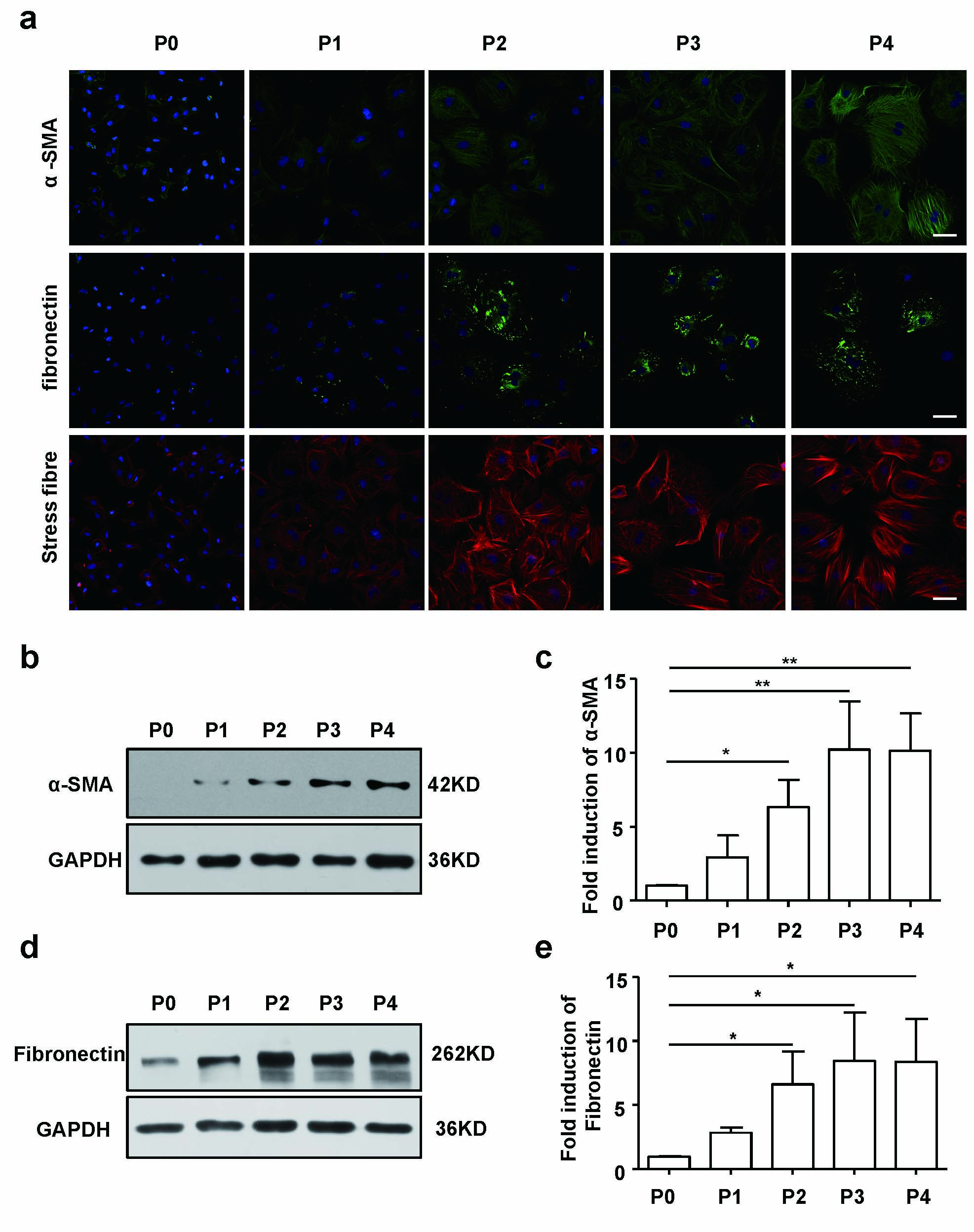
Figure S9.** The first-passage cardiac fibroblasts started to show the myofibroblast phenotype. (a) Different passages of cardiac fibroblasts were stained with α-SMA (green) or fibronectin (green) antibody or stress fibre (red), with rhodamine phalloidin. Scale bar: 50 μm. (b) & (c) The expression of α-SMA was determined and quantified *via* western blot analysis. N = 6 (d) & (e) The expression of fibronectin was determined and quantified *via* western blot analysis. N = 6. Data are shown as the mean ± S.E.M. * *P* < 0. 05 and ** *P* < 0. 01 using Kruskal–Wallis ANOVA combined with post hoc Dunn’s multiple comparison test.

**Author contributions**

Jing Shen performed the experiments, analysed the data and wrote the manuscript. Jiang-Hui Zhang performed the experiments and analysed the data. Han Xiao performed the experiments, analysed the data and wrote the manuscript. Ji-Min Wu performed the experiments and analysed the data. Kang-Min He analysed the data and revised the manuscript. Zhi-Zhen Lv interpreted the data. Zi-Jian Li interpreted the data and revised the manuscript. Ming Xu interpreted the data and revised the manuscript. You-Yi Zhang designed the study, interpreted the data and revised the manuscript.

In Figure 1, Jiang-Hui Zhang acquired the data, and Ji-Min Wu and You-Yi Zhang assembled the figure. In Figure 2, Jing Shen and Han Xiao acquired the data, and Kang-Min He and Zi-Jian Li analysed and assembled the data. In Figure 3, Jiang-Hui Zhang acquired the data, and Zhi-Zhen Lv and You-Yi Zhang analysed and assembled the data. In Figure 4, Jing Shen and Jiang-Hui Zhang acquired the data, and Ming Xu and You-Yi Zhang analysed and assembled the data. In Figure 5, Jing Shen, Jiang-Hui Zhang and Han Xiao acquired the data, and Ji-Min Wu and You-Yi Zhang analysed and assembled the data. In Figure 6, Jing Shen and Han Xiao acquired the data, and Ji-Min Wu and You-Yi Zhang analysed and assembled the data. In Figure S1, Jing Shen acquired the data, and Jiang-Hui Zhang and Han Xiao assembled the data. In Figure S2, Jing Shen and Jiang-Hui Zhang acquired the data, and Han Xiao assembled the data. In Figure S3, Jiang-Hui Zhang and Han Xiao acquired the data, and Ji-Min Wu and You-Yi Zhang analysed and assembled the data. In Figure S4, Jing Shen acquired the data, and Jiang-Hui Zhang and Han Xiao assembled the data. In Figure S5, Jing Shen acquired the data; Jiang-Hui Zhang and You-Yi Zhang analysed the data, and Zi-Jian Li assembled the data. In Figure S6, Han Xiao acquired the data, and Jiang-Hui Zhang and You-Yi Zhang analysed and assembled the data. In Figure S7 and S8, Jing Shen and Jiang-Hui Zhang acquired the data, and Han Xiao and You-Yi Zhang analysed and assembled the data. In Figure S9, Han Xiao acquired the data, and Jiang-Hui Zhang and You-Yi Zhang analysed and assembled the data.


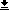

Supplement: Supplementary file 1 — supplemental data [file 41419_2017_145_MOESM1_ESM.docx]
